# The natural history of acute Ebola Virus Disease among patients managed in five Ebola treatment units in West Africa: A retrospective cohort study

**DOI:** 10.1371/journal.pntd.0005700

**Published:** 2017-07-19

**Authors:** Kelly Skrable, Reshma Roshania, Michaela Mallow, Vanessa Wolfman, Matthew Siakor, Adam C. Levine

**Affiliations:** 1 Warren Alpert Medical School of Brown University, Providence, Rhode Island, United States of America; 2 International Medical Corps, Los Angeles, California, United States of America; Institute of Tropical Medicine, BELGIUM

## Abstract

**Introduction:**

Previous studies of Ebola Virus Disease (EVD) have focused on clinical symptoms and Ebola virus (EBOV) cycle threshold (CT) values recorded at patient triage. Our study explores EVD symptoms and EBOV CT values from onset of illness to recovery or death in a diverse population of patients.

**Methodology/Principal findings:**

We analyzed clinical care data from EBOV positive patients admitted to five Ebola treatment units in West Africa from 2014–2015. Prevalence of clinical signs/symptoms and CT values were explored using descriptive statistics. Logistic regression was used to examine their association with mortality. Survival was analyzed using Kaplan-Meier estimators from symptom onset date to death. During the first week of illness, dyspnea (OR = 2.44, 95% CI: 1.07–5.85) and tachycardia (OR = 10.22, 95% CI: 2.20–56.71) were associated with higher odds of mortality. Dyspnea (OR = 2.33, 95% CI: 1.210–4.581), bleeding (OR = 2.51, 95% CI: 1.219–5.337), and diarrhoea (OR = 2.79, 95% CI: 1.171–6.970) at any point during the illness course were associated with higher odds of mortality. Higher initial (OR = 0.85, 95% CI: 0.81–0.89) and mean (OR = 0.60, 95% CI: 0.53–0.66) CT values were associated with lower odds of mortality. CT values reached their nadir after 3–5 days of illness and then rose in both survivors and non-survivors until recovery or death.

**Conclusions/Significance:**

Our study demonstrates the population prevalence of clinical signs/symptoms and EBOV CT values over time in a large, diverse cohort of patients with EVD, as well as associations between symptoms/EBOV CT values and mortality. These findings have implications on surveillance, operational planning, and clinical care for future EVD outbreaks.

## Author summary

Previous studies of Ebola Virus Disease (EVD) have focused on clinical symptoms and viral load (or its proxy of cycle threshold value) in the blood of patients measured the day they begin medical care. However, this provides a single snapshot of the illness in time and does not indicate how signs/symptoms and cycle threshold values change over time. This study examined EVD symptoms and the Ebola virus cycle threshold values in patients from the time the illness started to the time they recovered or died. The patients studied were admitted to five treatment units in West Africa over a one-year period. The authors found that the youngest (under five) and oldest (45 and older) patients were least likely to survive EBOV infection. In addition, higher levels of virus in the blood at the time patients sought treatment as well as higher virus levels over the duration of their illness were associated with lower survival. These findings provide important insight into the progression of EVD over time and will help clinicians and other health workers to identify and design interventions for subgroups of patients who are more likely to die from EBOV infection.

## Introduction

The West Africa Ebola Virus Disease (EVD) outbreak that began in Guinea in December 2013 escalated to become the largest and deadliest in history, with over 28,600 EVD cases and 11,300 deaths reported globally.[1, 2] Although the World Health Organization (WHO) initially declared that human-to-human transmission ended in Sierra Leone, Liberia and Guinea at various points in 2015, new cases of EVD, likely related to persistent virus in EVD survivors, continued to emerge in West Africa in 2016 during periods of enhanced surveillance.[2]

Prior studies have documented some aspects of EVD natural history.[3–9] A 2015 meta-analysis of ten studies of EVD outbreaks identified bleeding events, cough, sore throat, conjunctivitis, abdominal pain, vomiting, and diarrhoea as symptoms associated with increased mortality risk.[4] Studies of the recent West African outbreak identified weakness, extreme fatigue, vomiting, diarrhoea, confusion, bleeding, anorexia, dyspnea and fever as associated with increased mortality risk.[5–8, 10] Mortality risk was consistently found to be higher among older persons[5–9] and in patients with higher initial viral loads[7, 8] and lower real time polymerase chain reaction (RT-PCR) cycle threshold (CT) values (which are inversely proportional to viral load).[11–13]

Though the West African outbreak has expanded our EVD knowledge, there is still much we do not know about its natural history. Aside from a handful of case reports from the United States and Europe, prior studies of EVD natural history have generally focused on presenting symptoms and initial CT value/viral load, which provide a single snapshot in time and do not indicate how signs/symptoms and viral dynamics evolve over time. Most prior studies have also only included patients from a single site or a single phase of the epidemic, limiting their generalizability.

Using data collected as part of clinical care at five Ebola Treatment Units (ETUs) across Liberia and Sierra Leone over the course of a full year, this study aims to expand our understanding of the natural history of EVD from symptom onset to recovery or death, which can lead to improved surveillance and clinical management during future outbreaks.

## Methods

### Study design and patient population

This retrospective cohort study includes data from EBOV positive patients admitted to two ETUs in Liberia, in Bong and Margibi Counties, and three ETUs in Sierra Leone, located in Port Loko District, Bombali District, and Kambia District. The ETUs were operated by the humanitarian organization International Medical Corps (IMC) between September 15, 2014 and September 15, 2015.

The Sierra Leone Ethics and Scientific Review Committee, the University of Liberia—Pacific Institute for Research & Evaluation Institutional Review Board, and the Lifespan (Rhode Island Hospital) Institutional Review Board provided ethical approval and exemption from informed consent.

### Data collection

Patient demographic data were collected at admission. Trained nurses, physician assistants, or physicians recorded clinical sign/symptom data at least once daily on standardized clinical care paper forms. The data collection forms can be found in the S1 Appendix. All data were collected as part of routine clinical care and later combined into a unified research database, as described previously.[14] A summary of the standard clinical protocols for the ETUs can be found in the S2 Appendix.

In Liberia, the United States Naval Medical Research Center (NMRC) Mobile Laboratory conducted EBOV diagnostic testing for both IMC ETUs using the 1-step quantitative Ebola Zaire real-time reverse transcriptase–polymerase chain reaction (RT-PCR) (TaqMan) assay (Naval Medical Research Center, Frederick, MD). In this assay, QIAamp Viral RNA Mini Kit was used to extract Qiagen buffer AVL/ethanol-inactivated blood samples. Extracted ribonucleic acid was tested for two EBOV gene targets (EBOV Zaire locus and minor groove binding locus) using the Applied Biosystems StepOnePlus instrument. A sample was confirmed to be positive for EBOV if both targets were detected. If only one target was detected, the sample was considered indeterminate and the patient was retested.[14]

The Public Health England (PHE) laboratories in Port Loko and Bombali districts, Sierra Leone, performed laboratory EBOV testing for patients admitted to the ETUs in those districts. With support from the European Mobile laboratory, the Nigerian laboratory in Kambia district provided RT-PCR testing for patients admitted to the Kambia ETU in Sierra Leone. The PHE and Nigerian laboratories tested only a single EBOV gene target (EBOV Zaire locus) as opposed to two targets; otherwise, their processes were similar to those used by the NMRC laboratory as described above. Additionally, in February 2015 the PHE laboratories changed from using the commercially available Altona real time RT-PCR assay to the in-house Trombley assay.[14] All CT values presented in this study are based on RT-PCR of the same EBV Zaire locus using the RNA extraction procedures described above. A CT greater than 40 was considered negative in all cases.

### Variables of interest

Day of illness was calculated as the number of days from self-reported symptom onset date to the provider rounding date. Sign/symptom presence was self-reported and/or observed during rounding. Temperature was either measured orally or via an infrared thermometer depending on the resources available at the time in each location. Temperature values were recoded into a binary Fever variable using a cut-off of 38°C. Similar symptoms from different ETUs were combined into single variables (e.g. combining myalgias and arthralgias into one category) for analysis. Patients over five years of age were divided into 10–year age blocks through age 45 to explore the non-linear relationships between age and outcomes of interest. Children under five were grouped together as per World Health Organization guidelines recognizing this as a particularly vulnerable age group.[15] CT values were treated as a continuous variable for EBOV positive tests. EBOV negative tests (i.e. after patient recovery) were assigned a value equal to or greater than 40.0 since exact CT values were not recorded beyond this threshold. Outcome was categorized as Survived, Deceased, or Transferred.

### Data quality

To assess the quality of data entered from original patient charts into the site-specific databases, we employed Lot Quality Assurance Sampling (LQAS), a random sampling methodology. [14] We selected random samples of 19 patient ID numbers from two sub-strata, EBOV+ and EBOV-, from each ETU (except Margibi, where 19 total patient ID numbers were randomly selected because only five EBOV+ patients were admitted) to audit the quality of the data. The audit results indicated a high number of discrepancies between data on scanned patient charts and data in the database. We therefore reentered all triage data for admitted patients from the scanned original patient charts. Daily rounding and treatment data were reentered for EBOV positive patients only due to prioritization of limited resources.

Additional quality assurance strategies to ensure accuracy of the data reentry included utilizing data validation settings in Excel reentry documents, standardizing patient data coding from various types of patient charts using a codebook, conducting data audits by data entry research assistants, and discussing data entry concerns with the Principal Investigator to develop standardized solutions. A subsequent data quality audit of the reentered data utilizing LQAS found that approximately 99% of the data in International Medical Corps’ unified database were consistent with data from scans of patient charts.[14]

### Data analysis

We conducted independent samples t-tests and one-way analysis of variance (ANOVA) testing, as appropriate, to assess differences in days of symptoms prior to admission and initial and mean CT value by subgroup. We calculated percentages of patients reporting clinical signs/symptoms on each day of illness and plot this data over time separately for patients who survived and died. We calculated mean CT values for each day of illness and present them graphically for patients who survived and died using locally weighted regressions to smooth the curves. Since patients were admitted at different points in their illness, the overall numbers of patients providing clinical or laboratory data was calculated for each day of illness and presented in the x-axes of the plots of each figure.

We conducted multivariable logistic regressions to analyze the associations among baseline characteristics, signs/symptoms over the length of illness, initial and mean CT values, and mortality, and present the odds ratios and 95% confidence intervals (CI). Separate models were created for the presence of sign/symptoms at any point during illness as well as during the first week of illness. All symptoms and patient age were included in the models after assessing for multicollinearity. In addition, all laboratory models controlled for inter-laboratory differences in assays and techniques by including the laboratory where the test was performed as an independent predictor of the CT value. The odds ratios presented all reflect multivariable analyses; thus, significant findings are significant after controlling for all other covariates. Regression analyses were restricted to patients for whom data were available for key variables: outcome, triage date, symptom onset date, age category, and CT value for the CT analyses; missing data were excluded pairwise for other variables. Absence of symptom data was treated as absence of the symptom in all models. In the remaining tables, missing demographics (age and sex) were noted in the footnote and omitted from the frequency distributions.

Survival analyses were conducted using Kaplan-Meier estimates from symptom onset date to death; patients who were transferred were treated as censored observations while survivors were censored after 30 days of symptoms due to lack of long-term follow up data. Cox proportional hazard models were used to compare survival curves by subgroup. Statistical significance was established at 0.05. Data analyses were conducted in R version 3.2.1.

## Results

### Baseline demographics

**Fig 1:**
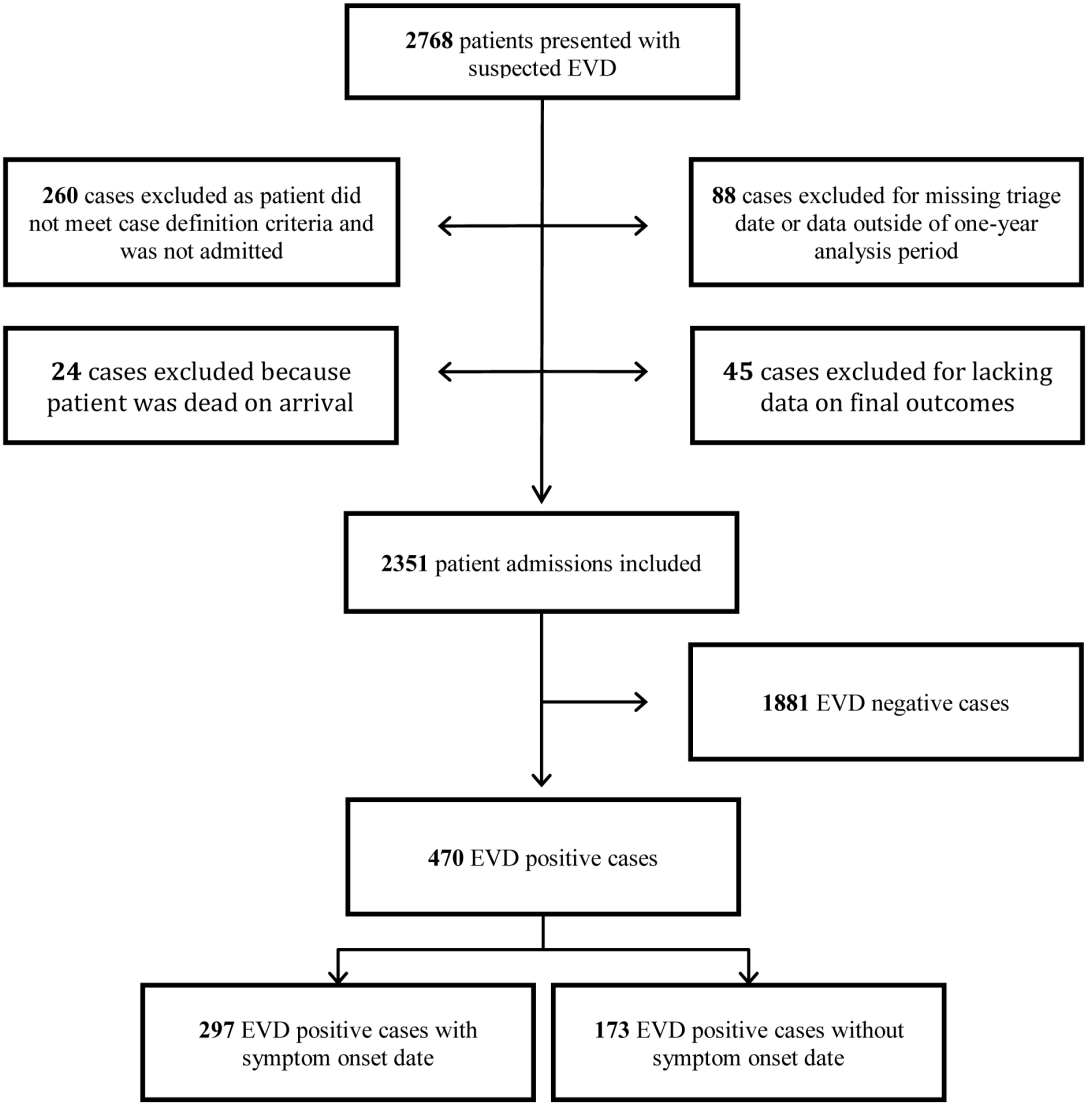
Study flow diagram.

The complete IMC dataset included data for 2,768 patients, of which 470 were EBOV positive patients admitted during the one-year study period with final outcome data available (Fig 1). Of these patients, 297 patients reported a symptom onset date, allowing for analysis of signs, symptoms, and EBOV CT values over time. We found no significant differences in sex (p = 0.14), age (p = 0.06), or mortality (p = 0.76) between those with and without available symptom onset dates. The mean number of days of symptoms prior to admission was 4.3 (IQR: 2.0, 6.0). This varied significantly by sex and age; however, we found no significant difference between mean days of symptoms and outcome (Table 1).

**Table 1:** Mean days of symptoms prior to ETU arrival for all patients and for individual subgroups.

	n (%)	Mean days of symptoms prior to ETU arrival	SD	p*
All	297 (100.0)	4.3	3.6	
Sex				0.0357
Male	127 (42.76)	4.8	4.2	
Female	170 (57.24)	3.9	3.0	
Age Category (years)				0.0086
0 to 4	21 (7.07)	2.4	1.5	
5 to 24	87 (29.29)	3.7	2.9	
25 to 44	118 (39.73)	4.6	3.9	
≥ 45	71 (23.91)	5.0	4.0	
Country				0.5604
Sierra Leone	123 (41.41)	4.1	3.9	
Liberia	174 (58.59)	4.4	3.4	
Outcome**				0.7655
Survived	122 (41.64)	4.4	4.3	
Deceased	171 (58.36)	4.2	3.1	

* t-test and ANOVA

**Transferred patients not included (n = 4)

**Fig 2:**
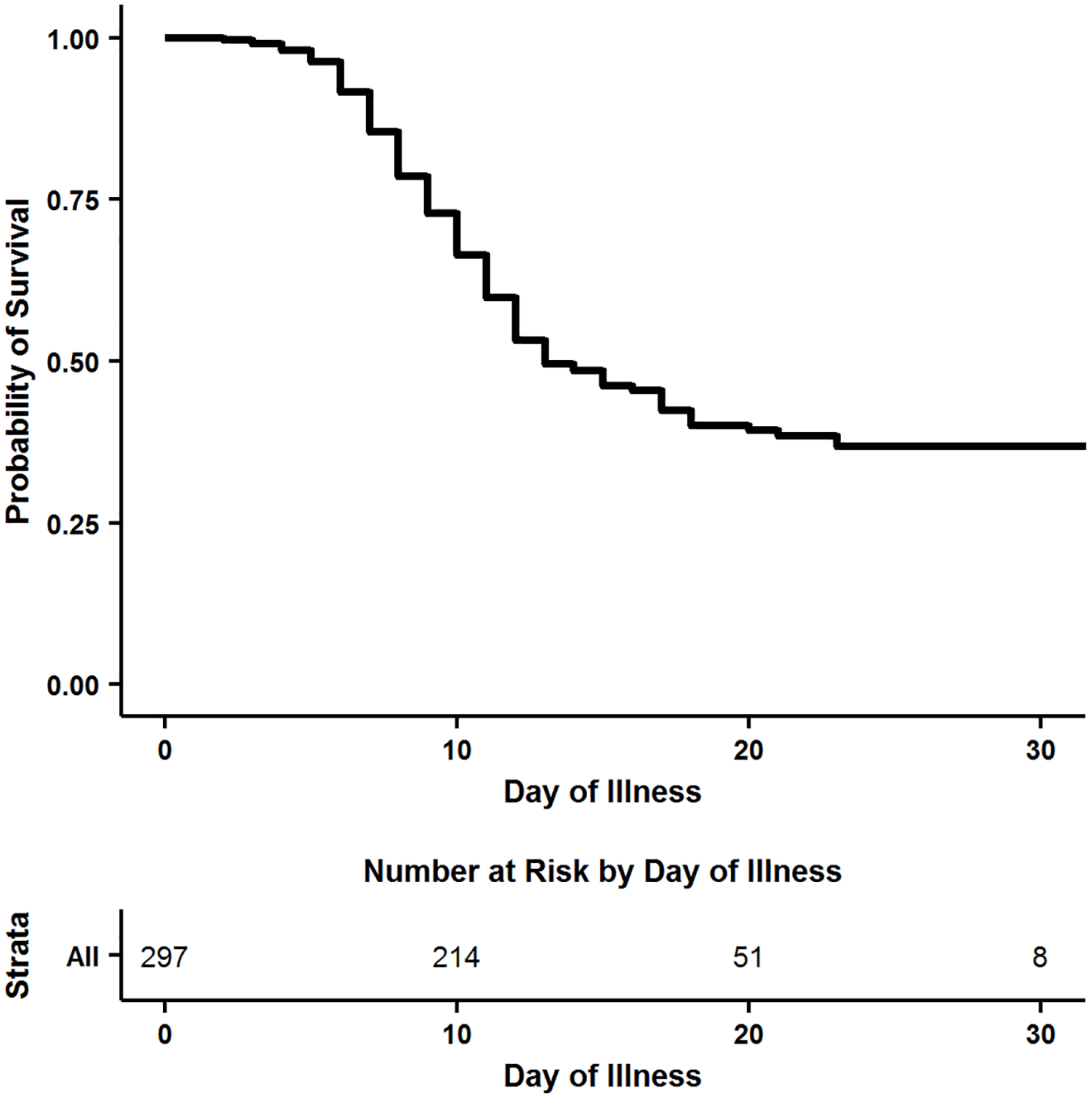
Kaplan-Meier survival analysis by symptom day for EBOV positive patients.

**Fig 3:**
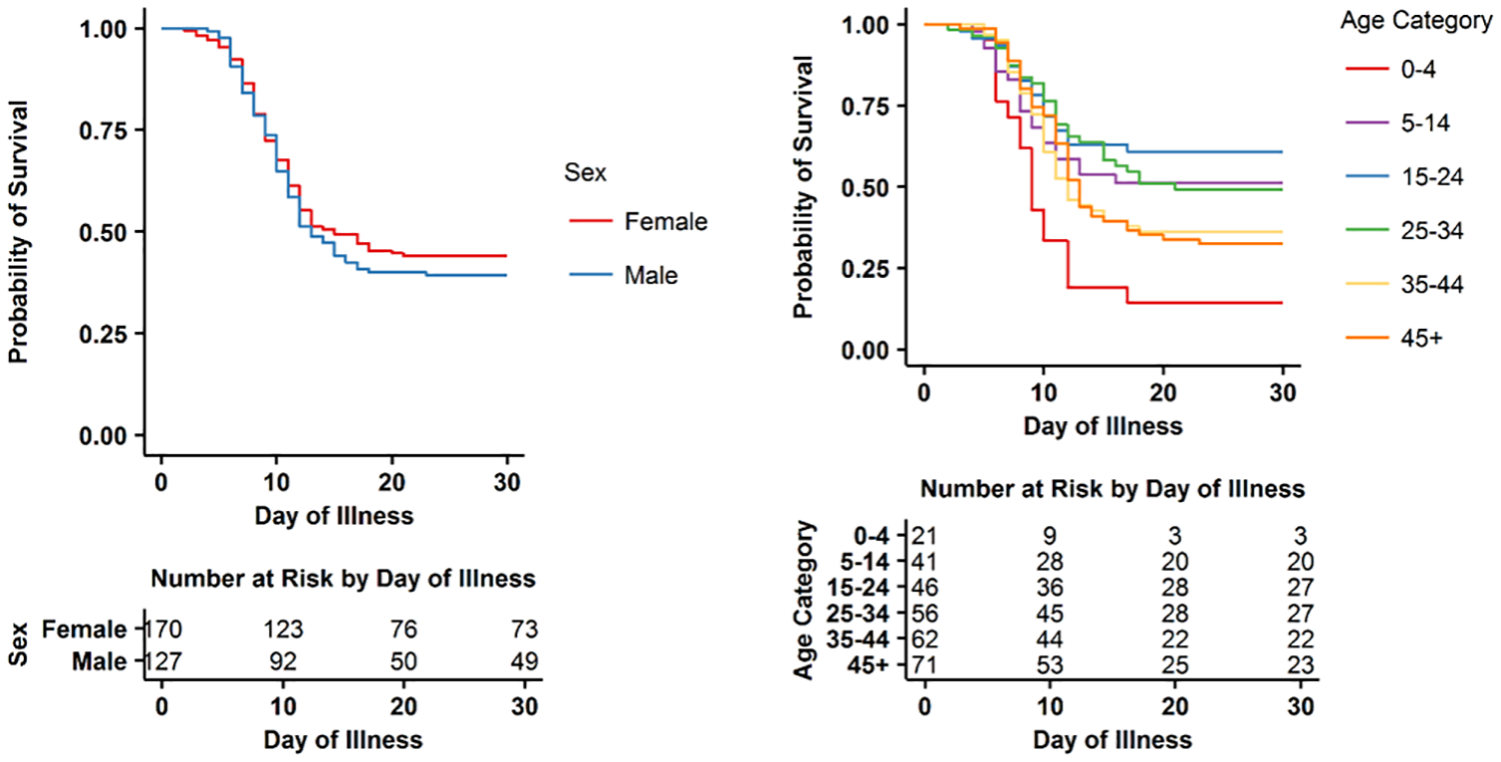
**A. Kaplan-Meier survival analysis by day of illness for EBOV positive patients disaggregated by sex. B. Kaplan-Meier survival analysis by day of illness for EBOV positive patients disaggregated by age**. *Variables have been abbreviated: Abdominal Pain = “Abd Pain”; Nausea/Vomiting = “Vomiting”; Myalgias/Arthralgias = “Myalgias”; Conjunctivitis = “Red Eyes”.

Fig 2 shows the Kaplan-Meier survival function for all patients based on day of illness. Among patients who died, mortality occurred in 88% of patients during their first two weeks of symptoms, and the last death in our sample occurred on the 23^rd^ day of illness. There was no statistically significant difference in survival by sex or by year (Fig 3A; Table 2). EBOV positive patients aged 15–24 had the highest overall probability of survival whereas children under five had the lowest probability of survival and patients over 45 had the second lowest probability of survival (Fig 3B; Table 2).

**Table 2:** Baseline demographics and mortality risk by demographic subgroup for EBOV positive patients with known symptom onset date.

	n (%)	Case Fatality Ratios* (%)	Mortality Risk from Survival Curves		
			Hazard Ratios	95% CI Lower	95% CI Upper
All	297 (100.0)	58.36	-	-	-
Sex*					
Male	127 (42.76)	60.80	1.12	0.83	1.51
Female	170 (57.24)	56.55	-	-	-
Age Category (years)**					
0 to 4	21 (7.07)	85.71	3.65	1.90	7.05
5 to 14	41 (13.80)	50.00	1.39	0.73	2.63
15 to 24	46 (15.49)	40.00	-	-	-
25 to 34	56 (18.86)	50.91	1.29	0.71	2.34
35 to 44	62 (20.88)	63.93	1.89	1.08	3.30
≥ 45	71 (23.91)	67.61	1.89	1.10	3.25
Country					
Sierra Leone	123 (41.41)	65.55	1.36	1.00	1.83
Liberia	174 (58.59)	53.45	-	-	-
Year					
2014	212 (72.35)	56.13	-	-	
2015	81 (27.65)	64.2	1.32	0.83	1.51

* Not including Transferred patients (n = 4)

** Missing values not included

### Signs and symptoms of EVD over time

**Fig 4:**
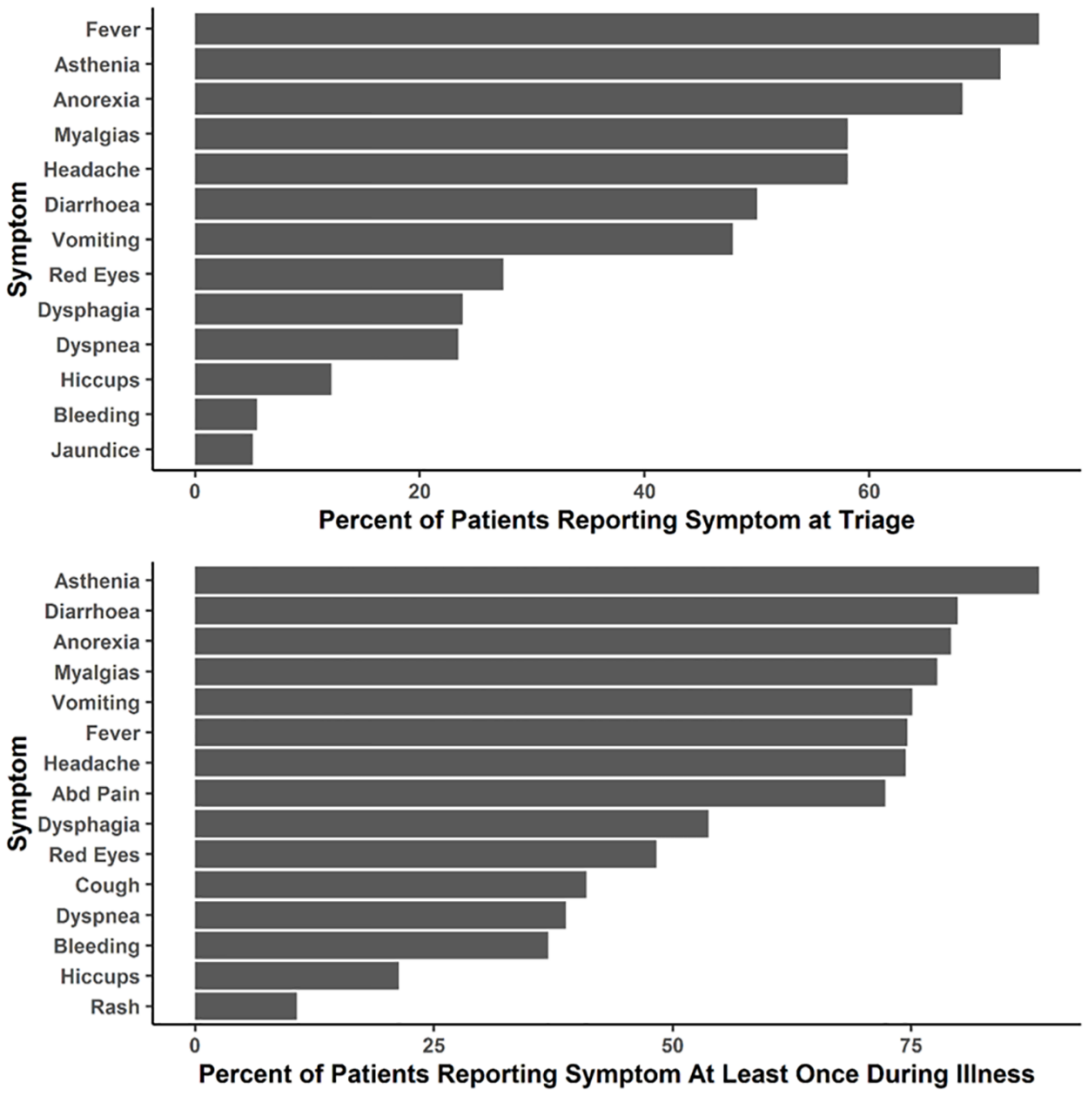
A. Signs/Symptoms at triage for all EBOV positive patients (n = 470)*. B. Signs/Symptoms over length of illness for all EBOV positive patients (n = 422). *Variables have been combined: Myalgias/Arthralgias = "Bone Pain", "Bone and Muscle Pain" and "Joint Pain"; Nausea/Vomiting = "Nausea" and "Vomiting"; Dysphagia = "Swallowing Problems" and "Sore Throat"; Conjunctivitis = "Red Injected Eyes" and "Conjunctivitis" Variables have been abbreviated: Abdominal Pain = “Abd Pain”; Nausea/Vomiting = “Vomiting”; Myalgias/Arthralgias = “Myalgias”; Conjunctivitis = “Red Eyes”.

Among all 470 patients with EVD, the most common signs/symptoms at triage were fever, asthenia (weakness), and anorexia (loss of appetite) (Fig 4A) whereas the most common signs/symptoms recorded at least once over the length of illness were weakness, anorexia, and diarrhoea (Fig 4B).

**Fig 5:**
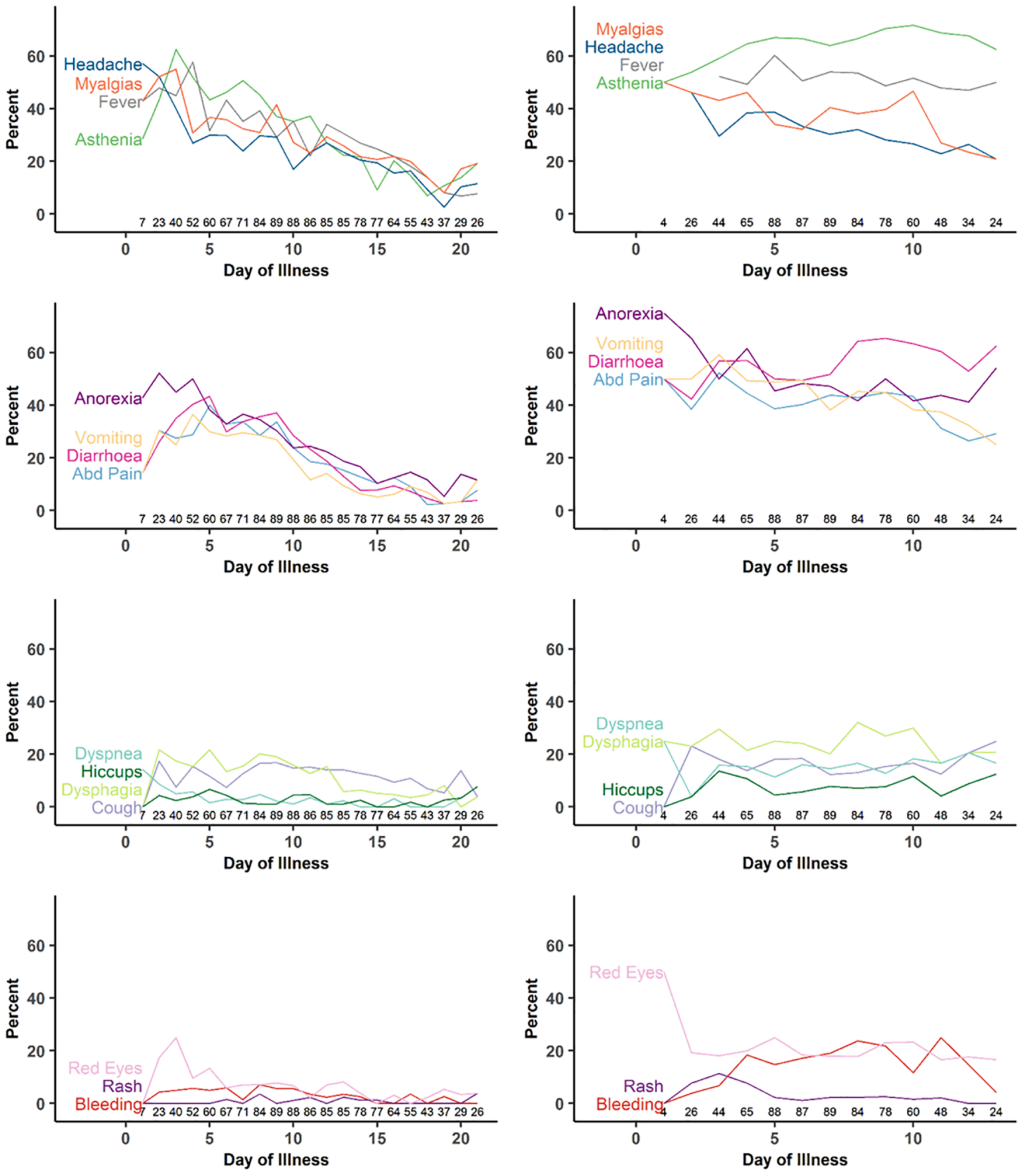
**(A-D). Symptoms by day of illness for surviving EBOV positive patients (E-H) symptoms by day of illness for non-surviving EBOV positive patients**. * Numbers along the x-axis indicate the number of patients providing data for that day. Variables have been abbreviated: Abdominal Pain = “Abd Pain”; Nausea/Vomiting = “Vomiting”; Myalgias/Arthralgias = “Myalgias”; Conjunctivitis = “Red Eyes”.

Fig 5 displays the daily population prevalence of symptoms from onset of illness to recovery or death among surviving and non-surviving patients with reported symptom onset dates and available data for that specific day. Among survivors (Fig 5A–5D), over half of patients reported headache and anorexia on the first day of illness. By the third day, fever, weakness, and myalgias/arthralgias increased, as did gastrointestinal symptoms, including stomach pain, nausea/vomiting, and diarrhoea. By the end of the first week of illness, the most commonly reported symptoms/signs were weakness, fever, and diarrhoea. The prevalence of most symptoms declined thereafter as patients recovered; common symptoms reported after three weeks of illness were mylagias/arthralgias and asthenia. Non-hemorrhagic rash and hiccups were among the least commonly reported signs/symptoms and remained so over the duration of illness. Among patients who died (Fig 5E–5H), over half of patients experienced weakness on each day of illness, and diarrhoea increased over the duration of illness.

Table 3 demonstrates the association of individual signs/symptoms with mortality. During the first week of illness, dyspnea (OR = 2.44, 95% CI: 1.07–5.85) and tachycardia (OR = 10.22, 95% CI: 2.20–56.71) were associated with higher odds of mortality. Over the entire length of illness, dyspnea (OR = 2.33, 95% CI: 1.21–4.58), bleeding (OR = 2.51, 95% CI: 1.22–5.34), and diarrhoea (OR = 2.79, 95% CI: 1.17–6.97) were associated with mortality. Myalgias/arthralgias were associated with reduced mortality over the entire length of illness (OR = 0.35, 95% CI: 0.12–0.93).

**Table 3:** Multivariable regression model for mortality based on signs/symptoms present at any time in the first week of illness and signs/symptoms present at any time over the total length of illness*.

	Total illness			Week one of illness		
	OR	2.50%	97.50%	OR	2.50%	97.50%
Abdominal Pain	2.06	0.89	4.91	1.12	0.52	2.41
Anorexia	0.99	0.34	2.89	1.17	0.42	3.21
Asthenia	1.78	0.50	6.34	1.19	0.43	3.28
Bleeding	2.51	1.22	5.34	2.01	0.77	5.52
Cough	0.38	0.20	0.71	0.82	0.39	1.73
Diarrhoea	2.79	1.17	6.97	1.31	0.59	2.96
Dyspnea	2.33	1.21	4.58	2.44	1.07	5.85
Fever	0.76	0.34	1.68	0.83	0.40	1.71
Headache	0.44	0.18	1.03	0.63	0.28	1.40
Hiccups	1.60	0.74	3.55	0.79	0.27	2.29
Myalgias/Arthralgias**	0.35	0.12	0.93	0.92	0.41	2.09
Nausea/Vomiting**	1.24	0.53	2.88	0.75	0.33	1.67
Non Hemorrhagic Rash	0·92	0.33	2.68	6.39	0.97	127.69
Conjunctivitis**	1.64	0.87	3.13	1.31	0.64	2.71
Dysphagia**	0.70	0.34	1.43	0.99	0.46	2.10
Tachycardia***	4.98	0.99	28.95	10.22	2.20	56.71
Total n = 297						

*Controlling for age category

**Variables have been combined:

Myalgias/Arthralgias = "Bone Pain", "Bone and Muscle Pain" and "Joint Pain"

Nausea/Vomiting = "Nausea" and "Vomiting"

Dysphagia = "Swallowing Problems" and "Sore Throat"

Conjunctivitis = "Red Injected Eyes" and "Conjunctivitis"

*** Heart rate > 100

### EBOV CT values over time

**Fig 6:**
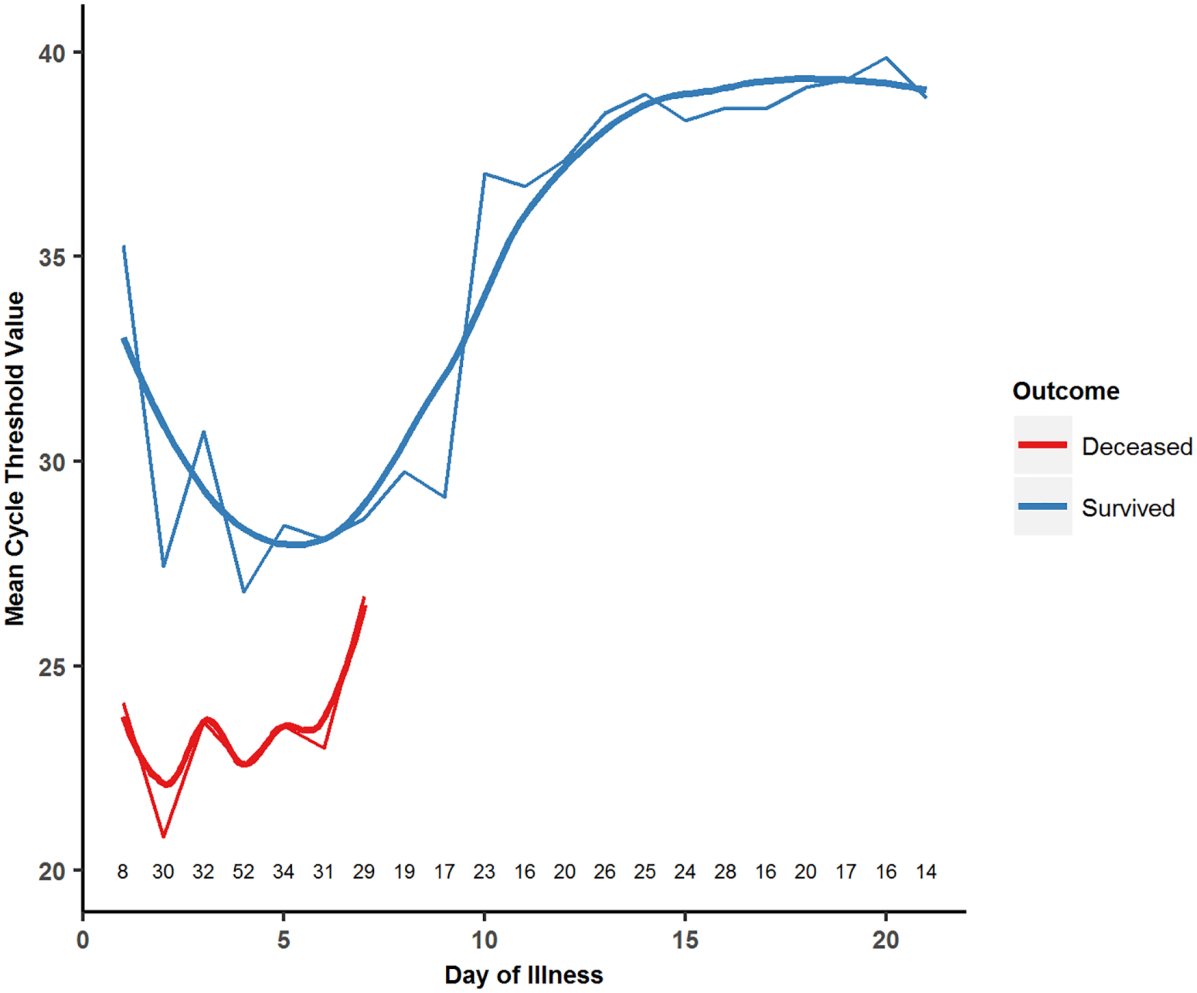
CT values by day of illness and final outcome for EBOV positive patients. *Numbers along x-axis indicate the number of patients providing data for that day.

349 patients had available CT values on arrival and 395 had available CT values at some point during their ETU stay. Fig 6 demonstrates the changes in CT values over time among patients with available symptom onset date and laboratory data for each day of illness, with separate curves drawn based on patient outcome. In both patients who survived and those who died, CT values decreased over the first three to five days, signifying a rapid increase in viral load. After this point, CT values begin to increase in both groups, signifying decreasing viral loads. Overall, patients who died had a lower initial CT value (recorded on ETU arrival) and mean CT value (recorded over their entire course of illness) compared to those who survived (Table 4). Additionally, patients who presented in 2014 had a higher initial CT value (26.56) than those who presented in 2015 (23.73, p = 0.0005) though there was no significant difference in mean CT values over the length of illness by year of presentation. In logistic regression analysis controlling for both age and the laboratory where testing was performed, both a higher initial CT value (OR = 0.85, 95% CI: 0.81–0.89) and a higher mean CT value (OR = 0.60, 95% CI: 0.53–0.66) were associated with a significantly lower likelihood of mortality.

**Table 4:** Initial cycle threshold (CT) values and mean CT values over entire length of illness for all EBOV positive patients and for individual demographic subgroups.

	n	Initial CT	SD	p	n	Mean CT	SD	p
All	349	25.43	7.37	-	395	32.67	8.44	-
Sex				0.0162				0.0346
Male	134	26.66	7.68		150	30.34	7.76	
Female	215	24.67	7.08		244	28.6	8.12	
Age Category (years)*				0.6524				0.0803
0 to 4	37	27.14	9.80		40	27.69	9.27	
5 to 14	48	24.98	7.20		56	30.1	7.57	
15 to 24	57	26.09	7.68		63	31.11	7.57	
25 to 34	65	25.32	6.96		77	30.22	7.66	
35 to 44	65	25.03	7.00		70	28.55	7.99	
≥ 45	77	24.84	6.57		88	27.87	8.12	
Outcome**				<0.0001				<0.0001
Survived	156	28.28	6.77		190	35.74	3.12	
Died	189	23.14	7.07		201	23.24	6.36	
Year								
2014	210	26.56	7.03		220	29.68	7.44	
2015	139	23.73	7.57		175	28.8	8.7	
				0.0005				0.2889

* Not including missing values

** Not including Transferred patients (n = 4)

## Discussion

This study is the first to describe the population prevalence of clinical signs/symptoms and CT values on each day of illness in a large cohort of patients infected with EVD who were managed in different countries at varying phases of the epidemic. In general, our study confirms the findings of several previous studies, while also demonstrating new findings and raising further questions for future research.

Our study demonstrates that nearly all signs/symptoms increase to their own peak in the first week of illness, followed by a slow decline thereafter in survivors and remaining relatively steady until death in non-survivors. Additionally, many patients developed symptoms over the course of their illness that were not present on ETU arrival, which would have been missed in previous research studies that only analyzed triage data. As in prior studies from both Africa and the United States/Europe, we found the most common signs/symptoms of EVD to be very non-specific, including headache, anorexia, myalgias/arthralgias, fever, weakness, and gastrointestinal symptoms.[4, 16–23] Less common signs/symptoms included dyspnea, conjunctivitis, dysphagia, non-hemorrhagic rash, hiccups, cough, and bleeding. While hemorrhage has been a prominent feature in previous outbreaks, we found bleeding to be one of the least frequently observed symptoms, present in less than ten percent of patients at triage and in about a third of patients at some point during their illness.[5, 6, 17, 24] These findings could be used to rationalize data collection and patient monitoring, helping providers plan for patient clinical care needs based on their anticipated symptom evolution as well as to help improve upon EVD case definitions in future outbreaks.

In our cohort, symptoms of bleeding, dyspnea, and diarrhoea were associated with increased mortality when present at any point during illness; however, among symptoms present during the first week of illness, only dyspnea and tachycardia were associated with increased mortality. Previously published research from this dataset also found that malaria co-infection was associated with higher mortality.[25] These findings are consistent with other research on the West African outbreak that sought to elucidate the predictors of mortality in EBOV positive patients.[5, 7, 10, 17] The most common causes of death in patients with EVD have been previously documented as hypovolemic shock and multisystem organ failure, both of which can result from the rapid fluid loss associated with severe diarrhoea and/or bleeding and may result in tachycardia and dyspnea.[10, 13] This highlights the critical importance of regular assessment of routine vital signs, including heart rate and respiratory rate, to identify those patients who may potentially be decompensating and most in need of aggressive intravenous volume repletion and/or other clinical interventions. Myalgias/arthralgias were associated with reduced mortality in our cohort when present at any point during illness. However, due to the retrospective nature of this dataset, it is difficult to elucidate whether myalgias/arthralgias were associated with improved survival only because less ill patients were more likely to live long enough to experience them and/or note their presence. Further research is needed to explore this finding.

Previous studies have correlated initial viral loads greater than 10^5^–10^6^ copies/mL with higher mortality and higher likelihood of more severe symptoms.[6, 7] Similarly, studies that utilized CT as a surrogate marker for viral load found that lower CT values in the first EBOV-positive blood sample corresponded to a higher likelihood of mortality.[11–13] Our findings were consistent with these previous studies, finding that both a lower initial CT value and a lower mean CT value were associated with a significantly higher likelihood of mortality.

In our study, CT values reached their nadir (equivalent to peak viral load) approximately five days after symptom onset and thereafter began to increase, with the most rapid rate of increase occurring approximately between days eight to twelve. Our data also demonstrates a notable reduction in signs/symptoms among survivors beginning at days eight to ten, corresponding temporally to the period of sharp increases in CT values. Fig 6 illustrates the changes in CT value by day of illness between survivors and non-survivors. As expected, the average CT values of those who died from EVD are lower than those of survivors at all points, indicative of persistently higher viremia in non-survivors, which is consistent with existing literature.[26] After reaching their respective nadirs, however, CT values begin to rise in both survivors and non-survivors. An immune response reducing viral load in both survivors and non-survivors may be a possible explanation for this observation.

Though we found few studies that analyzed changes in CT values or viral load over time from a large sample of EBOV positive patients, our data on viral kinetics are consistent with others that analyzed CT values over time.[26, 27] These studies also found that viral load was substantially higher in non-survivors than survivors at all time points during the course of illness, which mirrors the lower CT values in non-survivors compared to survivors in our cohort at all time points. Additionally, Lanini et al note that the viral load begins to fall in both survivors and non-survivors after reaching its peak, though the fall is more pronounced in survivors, which is identical to our results.[26] Lanini et al found that viral load peaked on day five in survivors, which is similar to our results; however, we found peak viral load in non-survivors to be earlier than Lanini.[26] Though the mean days of symptoms prior to seeking treatment is similar between our study (4.3 days) and that of Lanini et al (4.53 days), it is possible that potential recall bias in the self-reported date of symptom onset may have contributed to the discrepancies in peak viral load day found between the two studies. Further research is needed to explore the evolution of viral loads and other measures of immune response in both survivors and non-survivors.

The youngest patients (0–4 years) in our study had a significantly lower probability of survival when compared to all other age groups, followed by the oldest age group (45+). These findings of higher risk of mortality among children under five in this dataset are explored in more detail in a previous publication, which analyzed a subset of the same cohort and showed that among children aged <18, age <5 years and CT value ≤20 were both associated with significantly higher mortality. [28] The rapid fluid loss secondary to the severe vomiting and diarrhoea seen in EVD can cause dehydration, electrolyte imbalances, hypovolemic shock, and multisystem organ failure, especially in smaller children who have less blood volume or in older persons who are likely to have other preexisting health conditions. Further research is needed to analyze the vulnerabilities of the youngest and oldest patients, and targeted clinical management guidelines are needed to address the specific contributors to higher mortality in these age groups.

We found significant differences by sex and age in terms of days from symptom onset to ETU presentation. Sex was not associated with increased mortality, confirming previously published findings from the broader dataset.[14] Women arrived one day earlier than men on average and older patients presented later than younger patients. Women also represented a larger proportion of EBOV positive patients in our five ETUs, consistent with their role as traditional caretakers in rural households, putting them at greater risk of EBOV exposure. Further research is needed to explore the reasons for differences in health-seeking behavior to develop targeted health outreach messaging, as delays in identifying and isolating suspected cases can lead to increased disease dissemination.[17, 19]

### Limitations

These data were collected under extreme conditions in austere environments for purely clinical purposes. As such, objective measures of shock, such as blood pressure, were not collected in any of our ETUs due to the difficulty of using a stethoscope to measure blood pressure in full PPE and the difficulty in reusing equipment between patients, nor do we have consistent data across ETUs regarding renal, central nervous system, or pulmonary function. This limited the potential of this dataset to support more robust clinical analysis or draw inferences that would require such clinical variables. Symptom onset dates were generally self-reported (or reported by caregivers) and their accuracy may have been affected by recall bias. Signs/symptoms at triage and during ETU admission were either self-reported or recorded by clinicians with variable clinical training and experience. There may have been systematic underreporting among the youngest patients and those who were not able to communicate with health care providers. The data collection forms used across ETUs in Sierra Leone and Liberia also had minor differences in the clinical variables collected and in some instances data were aggregated into a more general symptom, thus losing analytic specificity. Because patients presented for treatment at different points in their illness, the total number of patients reporting symptoms varies by day of illness, as delineated in each figure, limiting our ability to construct true longitudinal models comparing symptoms and CT values over time in patient subgroups.

The ETUs used slightly different assays to extract viral RNA. Additionally, the assays used in Liberia analyzed two different gene loci for analysis, whereas assays used in Sierra Leone tested a single locus. The laboratories serving the Port Loko and Bombali districts in Liberia used an in-house assay from February 2015 onwards. The use of different laboratory assays across the ETUs may have affected the CT values presented, though we attempted to control for the testing laboratory in all of our analyses using laboratory data as described above. Our analysis is based on patients who agreed to seek treatment and survived long enough to reach ETUs for treatment and may therefore be susceptible to selection and survivor bias. The associations between mortality and various signs/symptoms may be affected by immortal time bias, though we addressed this by including an analysis of the association between signs/symptoms present in the first week of illness and mortality. In addition, long-term follow-up data are not available for our surviving patients. Finally, about a dozen of the patients included in this analysis received an experimental anti-viral treatment (ZMapp) as part of a separate trial, which may have impacted their outcomes.[29]

### Conclusion

Our study demonstrates changes in the population prevalence of clinical signs/symptoms and CT values over time in a large and diverse cohort of patients infected with EVD, as well as important differences in overall mortality by age group. These findings have implications for epidemiological surveillance and clinical care by helping to identify subpopulations at higher risk of morbidity and mortality; EVD case definition refinement by demonstrating the most common symptoms at various points of illness; operational planning for health outreach activities and clinical treatment; and survivor care for future EVD outbreaks. However, limitations in this retrospectively collected dataset highlight the need for future prospective studies during EVD outbreaks that include both clinical and biological data collection in order to improve upon the currently available data. Further research is needed to understand the differences in immune response between survivors and non-survivors and their implications for clinical care and the development of new vaccines and treatments for this devastating disease.

## Supporting information

S1 Appendix International Medical Corps EVD clinical data collection forms. (PDF)

S2 Appendix International Medical Corps standard clinical/psychosocial procedures for Ebola Treatment Unit (ETU) operations. (PDF)

S1 Checklist STROBE checklist. (DOC)
