# Supplementary material for: The natural history of acute Ebola Virus Disease among patients managed in five Ebola treatment units in West Africa: A retrospective cohort study
Source: PLoS Negl Trop Dis. 2017 Jul 19;11(7):e0005700. doi: 10.1371/journal.pntd.0005700 (PMC5536498; doi:10.1371/journal.pntd.0005700)
Supplement: S1 Appendix — (PDF) [file pntd.0005700.s001.pdf]

S1 Appendix. Table of Contents

Liberia Forms 2

Viral Hemorrhagic Fever Case Investigation Form 2

Rounding Form 6

Treatment Form 7

Laboratory Form 8

Sierra Leone Forms 9

Triage Form 9

Patient Tracking Form 11

Drug Request Form 12

Lab Request Form 13

Rounding Form 14

Nurse Inpatient Rounding Form 16

Treatment Form 17

Discharge Form 19

LIBERIA VIRAL HEMORHAGIC FEVER CASE INVESTIGATION FORM

Outbreak Case ID: ____________

Health Facility Case ID: ____________

Date of Case Report: ____/____/____ (D, M, Yr)

Section 1. Patient Information

Patient's Surname: ____________ Other Names: ____________ Age: ______ ☐ Years ☐ Months

Gender: ☐ Male ☐ Female Phone Number of Patient/Family Member: ____________ Owner of Phone: ____________

Status of Patient at Time of This Case Report: ☐ Alive ☐ Dead *If dead,* Date of Death: ___/___/____ (D, M, Yr)

Permanent Residence:

Head of Household: ____________ Village/Town: ____________

Country of Residence: ____________ County: ____________ District: ____________

Occupation:

☐ Farmer ☐ Butcher ☐ Hunter/trader of game meat ☐ Miner ☐ Religious leader ☐ Housewife ☐ Pupil/student ☐ Child

☐ Businessman/woman; type of business: ____________ ☐ Transporter; type of transport: ____________

☐ Healthcare worker; position: ____________ healthcare facility: ____________ ☐ Traditional/spiritual healer

☐ Other; please specify occupation: ____________

Location Where Patient Became Ill:

Village/Town: ____________ County: ____________ District: ____________

GPS Coordinates at House: latitude: ____________ longitude: ____________

If different from permanent residence, Dates residing at this location: ___/___/____ - ___/___/____ (D, M, Yr)

Section 2. Clinical Signs and Symptoms

Date of Initial Symptom Onset: ____/____/____ **(D, M, Yr)**

Please tick an answer for ALL symptoms indicating if they occurred during this illness between symptom onset and case detection:

Fever ☐ Yes ☐ No ☐ Unk

If yes, Temp: ____° C Source: ☐ Axillary ☐ Oral ☐ Rectal

Vomiting/nausea ☐ Yes ☐ No ☐ Unk

Diarrhea ☐ Yes ☐ No ☐ Unk

Intense fatigue/general weakness ☐ Yes ☐ No ☐ Unk

Anorexia/loss of appetite ☐ Yes ☐ No ☐ Unk

Abdominal pain ☐ Yes ☐ No ☐ Unk

Chest pain ☐ Yes ☐ No ☐ Unk

Muscle pain ☐ Yes ☐ No ☐ Unk

Joint pain ☐ Yes ☐ No ☐ Unk

Headache ☐ Yes ☐ No ☐ Unk

Cough ☐ Yes ☐ No ☐ Unk

Difficulty breathing ☐ Yes ☐ No ☐ Unk

Difficulty swallowing ☐ Yes ☐ No ☐ Unk

Sore throat ☐ Yes ☐ No ☐ Unk

Jaundice (yellow eyes/gums/skin) ☐ Yes ☐ No ☐ Unk

Conjunctivitis (red eyes) ☐ Yes ☐ No ☐ Unk

Skin rash ☐ Yes ☐ No ☐ Unk

Hiccups ☐ Yes ☐ No ☐ Unk

Pain behind eyes/sensitive to light ☐ Yes ☐ No ☐ Unk

Coma/unconscious ☐ Yes ☐ No ☐ Unk

Confused or disoriented ☐ Yes ☐ No ☐ Unk

Unexplained bleeding from any site ☐ Yes ☐ No ☐ Unk

If Yes:

Bleeding of the gums ☐ Yes ☐ No ☐ Unk

Bleeding from injection site ☐ Yes ☐ No ☐ Unk

Nose bleed (epistaxis) ☐ Yes ☐ No ☐ Unk

Bloody or black stools (melena) ☐ Yes ☐ No ☐ Unk

Fresh/red blood in vomit (hematemesis) ☐ Yes ☐ No ☐ Unk

Digested blood/"coffee grounds" in vomit ☐ Yes ☐ No ☐ Unk

Coughing up blood (hemoptysis) ☐ Yes ☐ No ☐ Unk

Bleeding from vagina, other than menstruation ☐ Yes ☐ No ☐ Unk

Bruising of the skin (petechiae/ecchymosis) ☐ Yes ☐ No ☐ Unk

Blood in urine (hematuria) ☐ Yes ☐ No ☐ Unk

Other hemorrhagic symptoms ☐ Yes ☐ No ☐ Unk

If yes, please specify: ____________

Other non-hemorrhagic clinical symptoms: ☐ Yes ☐ No ☐ Unk

If yes, please specifiy: ____________

Section 3. Hospitalization Information

At the time of this case report, is the patient hospitalized or currently being admitted to the hospital? ☐ Yes ☐ No

If yes, Date of Hospital Admission: ____/____/____ (D, M, Yr) Health Facility Name: ____________

Village/Town: ____________ County: ____________ District: ____________

Is the patient in isolation or currently being placed there? ☐ Yes ☐ No *If yes,* date of isolation: ____/____/____ (D, M, Yr)

Was the patient hospitalized or did he/she visit a health clinic previously for this illness? ☐ Yes ☐ No ☐ Unk

If yes, please complete a line of information for each previous hospitalization:

Dates of Hospitalization	Health Facility Name	Village	County	Was the patient isolated?
__/__/__ - __/__/__ (D, M, Yr)				☐ Yes ☐ No
__/__/__ - __/__/__ (D, M, Yr)				☐ Yes ☐ No

Case Name: | | Outbreak Case ID: |

Section 4. Epidemiological Risk Factors and Exposures

IN THE PAST ONE(1) MONTH PRIOR TO SYMPTOM ONSET:

1. Did the patient have contact with a known or suspect case, or with any sick person before becoming ill? ☐ Yes ☐ No ☐ Unk

If yes, please complete one line of information for each sick source case:

Name of Source Case	Relation to Patient	Dates of Exposure (D, M, Yr)	Village	County	Was the person dead or alive ?	Contact Types**
		__/__/__ - __/__/__			☐ Alive<br>☐ Dead, date of death: __/__/__ (D, M, Y)	
		__/__/__ - __/__/__			☐ Alive<br>☐ Dead, date of death: __/__/__ (D, M, Y)	
		__/__/__ - __/__/__			☐ Alive<br>☐ Dead, date of death: __/__/__ (D, M, Y)	

****Contact Types: (list all that apply)**
1 – Touched the body fluids of the case (blood, vomit, saliva, urine, feces)
2 – Had direct physical contact with the body of the case (alive or dead)
3 – Touched or shared the linens, clothes, or dishes/eating utensils of the case
4 – Slept, ate, or spent time in the same household or room as the case

2. Did the patient attend a funeral before becoming ill? ☐ Yes ☐ No ☐ Unk

If yes, please complete one line of information for each funeral attended:

Name of Deceased Person	Relation to Patient	Dates of Funeral Attendance (D, M, Yr)	Village	County	Did the patient participate (carry or touch the body)?
		__/__/__ - __/__/__			☐ Yes ☐ No
		__/__/__ - __/__/__			☐ Yes ☐ No

3. Did the patient travel outside their home or village/town before becoming ill? ☐ Yes ☐ No ☐ Unk

If yes, Village: ______ County: ______ Date(s): __/__/__ - __/__/__ (D, M, Yr)

4. Was the patient hospitalized or did he/she go to a clinic or visit anyone in the hospital before this illness? ☐ Yes ☐ No ☐ Unk

If yes, Patient Visited: ______ Date(s): __/__/__ - __/__/__ (D, M, Yr)

Health Facility Name: ______ Village: ______ County: ______.

5. Did the patient consult a traditional/spiritual healer before becoming ill? ☐ Yes ☐ No ☐ Unk

If yes, Name of Healer: ______ Village: ______ County: ______ Date: __/__/__ (D, M, Yr)

6. Did the patient have direct contact (hunt, touch, eat) with animals or uncooked meat before becoming ill? ☐ Yes ☐ No ☐ Unk

If yes, please tick all that apply:

Animal:	Status (check one only):
☐ Bats or bat feces/urine	☐ Healthy ☐ Sick/Dead
☐ Primates (monkeys)	☐ Healthy ☐ Sick/Dead
☐ Rodents or rodent feces/urine	☐ Healthy ☐ Sick/Dead
☐ Pigs	☐ Healthy ☐ Sick/Dead
☐ Chickens or wild birds	☐ Healthy ☐ Sick/Dead
☐ Cows, goats, or sheep	☐ Healthy ☐ Sick/Dead
☐ Other; *specify*______	☐ Healthy ☐ Sick/Dead

7. Did the patient get bitten by a tick in the past 2 weeks? ☐ Yes ☐ No ☐ Unk

Section 6. Case Report Form Completed by:

Name: ______ Phone: ______ E-mail: ______

Position: ______ County: ______ Health Facility: ______

Information provided by: ☐ Patient ☐ Proxy; *If proxy*, Name: ______ Relation to Patient: ______

Case Name: ______

Cutbreak Case ID: ______

***If the patient is deceased or has already recovered from illness, please fill out the next section.*
***If the patient is currently admitted to the hospital, leave the next section blank (it will be completed upon discharge)*

Section 7. Patient Outcome Information

Please fill out this section at the time of patient recovery and discharge from the hospital OR at the time of patient death.

Date Outcome Information Completed: ____/____/______ (D, M, Yr)

Final Status of the Patient: ☐ Alive ☐ Dead

Did the patient have signs of unexplained bleeding at any time during their illness? ☐ Yes ☐ No ☐ Unk
If yes, please specify: ______

If the patient has recovered and been discharged from the hospital:

Name of hospital discharged from: ______ County: ______
If the patient was isolated, Date of discharge from the isolation ward: ____/____/______ (D, M, Yr)
Date of discharge from the hospital: ____/____/______ (D, M, Yr)

If the patient is dead:

Date of Death: ____/____/______ (D, M, Yr)
Place of Death: ☐ Community ☐ Hospital: ______ ☐ Other: ______
Village: ______ County: ______ District: ______

Date of Funeral/Burial: ____/____/______ (D, M, Yr) Funeral conducted by: ☐ Family/community ☐ Outbreak burial team
Place of Funeral/Burial:
Village: ______ County: ______ District: ______

Please tick an answer for ALL symptoms indicating if they occurred at any time during this illness including during hospitalization:

Fever	☐ Yes ☐ No ☐ Unk
If yes, Temp: ____° C Source: ☐ Axillary ☐ Oral ☐ Rectal	
Vomiting/nausea	☐ Yes ☐ No ☐ Unk
Diarrhea	☐ Yes ☐ No ☐ Unk
Intense fatigue/general weakness	☐ Yes ☐ No ☐ Unk
Anorexia/loss of appetite	☐ Yes ☐ No ☐ Unk
Abdominal pain	☐ Yes ☐ No ☐ Unk
Chest pain	☐ Yes ☐ No ☐ Unk
Muscle pain	☐ Yes ☐ No ☐ Unk
Joint pain	☐ Yes ☐ No ☐ Unk
Headache	☐ Yes ☐ No ☐ Unk
Cough	☐ Yes ☐ No ☐ Unk
Difficulty breathing	☐ Yes ☐ No ☐ Unk
Difficulty swallowing	☐ Yes ☐ No ☐ Unk
Sore throat	☐ Yes ☐ No ☐ Unk
Jaundice (yellow eyes/gums/skin)	☐ Yes ☐ No ☐ Unk
Conjunctivitis (red eyes)	☐ Yes ☐ No ☐ Unk
Skin rash	☐ Yes ☐ No ☐ Unk
Hiccups	☐ Yes ☐ No ☐ Unk
Pain behind eyes/sensitive to light	☐ Yes ☐ No ☐ Unk
Coma/unconscious	☐ Yes ☐ No ☐ Unk
Confused or disoriented	☐ Yes ☐ No ☐ Unk

Other non-hemorrhagic clinical symptoms: ☐ Yes ☐ No ☐ Unk
If yes, please specifiy: ______

Final Diagnosis

Confirmed __	Suspect __	Probable __	Negative __
If not Ebola, what is the diagnosis?			

Final Outcome

Died __	Discharged __	Transferred __	Fled __
In case of death, who performed the burial? ___________________________			
Comments:			

Formulary

Amoxacillin: Children: 20mg/kg PO twice daily for 5 days.
Cefixime: Adults 400mg PO once daily for 5 days. Children: 8mg/kg solution PO once daily for 5 days.
Ceftriaxone: Adults: 1000mg IV once daily for five days. Children: 50mg/kg IV once daily for 5 days.
Chlorpromazine: Adults: 50mg PO three times daily as needed.
Ciprofloxacin: Adults: 500mg PO twice daily for five days.
Coartem:

a. Adults (>35kg): 4 tablets by mouth twice daily for 3 days
b. Children (25-34kg): 3 tablets by mouth twice daily for 3 days
c. Children (15-24kg): 2 tablets by mouth twice daily for 3 days
d. Children (5-14kg): 1 tablet by mouth twice daily for 3 days

Haloperidol: Adults: 5mg IV as needed.
Diazepam: Adults: 5mg by mouth 3 times a day as needed.
Lorazepam: Adults: 1mg IV as needed. Children: 0.05mg/kg IV as needed.
Metoclopramide: Adults 10mg PO/IV 4 times daily for nausea. Children: 0.1mg/kg PO/IV 4 times daily for nausea.
Morphine Sulfate: Adults: 10mg by mouth 4 times per day as needed (can be increased to 20mg or even 30mg if needed for pain). Children: morphine sulfate 0.3mg.kg by mouth 4 times per day as needed for pain (can be increased to 0.5mg/kg).
Morphine hydrochloride: 0.1mg/kg IV 4 times per day as needed for both adults and children
Omeprazole: Adults + Children > 20kg: 20mg by mouth once daily. Children < 20kg: omeprazole 10mg (half capsule) by mouth once daily (mix in water)
ORS: Adults: 1.5 liters per day of ORS by mouth. Children: 40cc/kg per day of ORS by mouth. Extra ORS for some dehydration: Adults: 3 liters of ORS by mouth over 4 hours. Children 5-14 years: 1.5 liters of ORS by mouth over 4 hours. Children < 5 years: 50ml/kg of ORS by mouth over 4 hours
Paracetomol: Adults: 1 gram by mouth 4 times daily. Children: 15mg/kg by mouth 4 times daily.
Vitamin A: > 12 mo: 200,000 IU by mouth once daily on days 1, 2, 8. Children 6-12mo: 100,000 IU by mouth once daily on days 1, 2, 8.
Vitamin C: Adults: 500mg by mouth 3 times per day. Children: 250mg by mouth 3 times per day.
Zinc sulfate: > 6 months: zinc sulfate 20mg tab once daily for 10 days. < 6 months: zinc sulfate 10mg tab once daily for 10 days.

Day 1 = day of admission. Check all symptoms that apply for each indicated day.

Day:	1	2	3	4	5	6	7	8	9	10	11	12	13	14
**General symptoms**														
Fever														
Fatigue / asthenia / weakness														
Muscle pain / Joint pain														
Headache														
Non-haemorrhagic rash														
Hiccups														
Anorexia (loss of appetite)														
Nausea														
Vomiting														
Dehydration														
Oedema														
Sore throat / Dysphagia														
Abdominal pain														
Abdominal tenderness														
RUQ pain														
Diarrhea (non-bloody)														
Hepatomegaly														
Splenomegaly														
Jaundice														
Anuria (unable to urinate)														
Dyspnea (breathlessness)														
Cough														
Chest pain														
Back pain														
Disorientation														
Red eyes / conjunctivitis														
General other 1: …………………														
General other 2: ………………….														
General other 3: ………………….														

Day:	1	2	3	4	5	6	7	8	9	10	11	12	13	14
**Haemorrhagic symptoms**														
Petechiae / cutaneous bruising														
Bleeding at injection site														
Bleeding gums														
Bloody/black diarrhea														
Haemetemesis (bloody vomit)														
Epistaxis (nose bleeds)														
Abnormal vaginal bleeding														
Haemoptysis (coughing blood)														
Haematoma (internal bleeding)														
Haematuria (bloody urine)														
Conjunctival Haemorrhage (bruising in white part of eyes)														
Bloody other 1: …………………														
Bloody other 2: …………………														

Patient Name: ______________________________ Age: ______ Sex: ____ ID Number:____________

Weight: _________kg (children only)

**Vital Signs**<br>Date	_____/_____				_____/_____				_____/_____				_____/_____			
Shift	AM	AFT	EVE	NITE	AM	AFT	EVE	NITE	AM	AFT	EVE	NITE	AM	AFT	EVE	NITE
Day	1	1	1	1	2	2	2	2	3	3	3	3	4	4	4	4
Temperature ºC (axillary)																
Heart rate / pulse (beats/minute)																
Respiratory rate (breath/minute)																
**Treatment: Doctors write in dosage and route under medication name (see formulary), then place circles for when to give medication. Nurses mark an X through the circle when medication is given.**																
ORS	O	O	O		O	O	O		O	O	O		O	O	O	
Cefixime	O				O				O				O			
Coartem	O		O		O		O		O		O					
Paracetamol	O	O	O	O	O	O	O	O	O	O	O	O	O	O	O	O
Omeprazole	O				O				O				O			
Vitamin A	O				O											
Vitamin C	O	O	O		O	O	O		O	O	O		O	O	O	
Extra ORS																
Ringers Lactate																
Morphine sulfate																
Morphine HCL																
Metoclopromide																
Zinc Sulfate																
Diazepam																
Lorazepam																
Chlorpromazine																
Halperidol																
Ceftriaxone																
Ciprofloxacin																

Patient Name: ______________________________ Age: ______ Sex: ____ ID Number:____________

Outbreak Case ID: ____________

LABORATORY FORM

Patient's Surname: ______________________ Other Names: ____________________________

Age: _______ ☐ Years ☐ Months

Gender: ☐ Male ☐ Female

Permanent Residence:

Village/Town: ______________________ County: ___________________________ Country of Residence: ________________

Date of Initial Symptom Onset: ____/____/______ **(D, M, Yr)**

Status of Patient at Time Sample Collected: ☐ Alive ☐ Dead *If dead,* Date of Death: ___/___/____ (D, M, Yr)

Health Facility Submitting Sample: ________________________ **Person Submitting Sample:** __________________________

Section 5. Clinical Specimens and Laboratory Testing

Specimen/shipping instructions:

- Label sample with **patient name, date of collection,** and **case ID**
- Send sample **cold** with a **cold/ice pack,** and **packaged appropriately**.
- Collect whole blood in a purple top (EDTA) tube – green or red top tubes acceptable if purple not available
- **Preferred sample volume = 4ml** (minimum sample volume = 2ml)

Has this patient had a sample submitted previously? ☐ Yes ☐ No

Sample 1: *Do not complete UVRI Only*

Sample Collection Date: ____/____/______ (D, M, Yr)

Sample Type:

☐ Whole Blood
☐ Post-mortem heart blood
☐ Skin biopsy
☐ Other specimen type, specify: ________________

Sample 2: *Do not complete UVRI Only*

Sample Collection Date: ____/____/______ (D, M, Yr)

Sample Type:

☐ Whole Blood
☐ Post-mortem heart blood
☐ Skin biopsy
☐ Other specimen type, specify: ________________

TRIAGE FORM – INTERNATIONAL MEDICAL CORPS – LUNSAR EBOLA TREATMENT CENTRE

DATE: ____ / ____ / 20__ DD / MM / YYYY	PATIENT ID #: LU–☐ – ☐☐☐☐☐ LU-2 = Triage; LU-3 = Confirmed; LU-4 = morgue

Form completed by (write your name): ______________________________________

Where did patient come from? ☐Ambulance ☐Referral ☐Walk-in

Where is the patient being triaged? ☐Ambulance/Community ☐ETC Triage tent

BASIC PATIENT INFORMATION

Information provided by: ☐Patient ☐ Someone else

If Someone else: Relation to patient: ____________________

Patient name: Surname ______________________ First name ______________________

Estimated age: ☐ ☐YEARS ☐MONTHS (for children under 1 year)

Sex: ☐Male ☐Female **Height:**______cm **Weight:**______kg **Z-Score:**________

Address: District _______________ Chiefdom_____________ Town/village ____________

SYMPTOMS & CONTACT HISTORY

Date when symptoms started: ________________________________

**Fever**	Yes	No	**# days:**_____ **Temperature:** ___________ ºc		
**Headache**	Yes	No			
**Nausea**	Yes	No			
**Vomit**	Yes	No	**Bloody**	Yes	No
**Diarrhoea**	Yes	No	**Bloody**	Yes	No
**Haemorrhagic eyes**	Yes	No			
**Other haemorrhage**	Yes	No	**Location**:		
**Breathlessness**	Yes	No			
**Bone/muscle pain**	Yes	No			
**Loss of appetite**	Yes	No			
**Asthenia/weakness**	Yes	No			
**Abdominal pain**	Yes	No			
**Jaundice**	Yes	No			
**Swallowing problems**	Yes	No			
**Hiccups**	Yes	No			

**Contact History Last 21 Days**	**(List contacts in contact tracing form)**	
Is there somebody ill in the family?	Yes	No
Have you visited someone who is ill?	Yes	No
Has somebody died recently in your family?	Yes	No
Have you been to a funeral recently?	Yes	No

Suspicion of EBOLA Yes No Ward/Bed # Admitted into______________

DATE: ____ / ____ / 20__
DD / MM / YYYY

PATIENT ID #: LU–[ ]–[ ][ ][ ][ ][ ]
LU-2 = Triage; LU-3 = Confirmed; LU-4 = morgue

ADDITIONAL PATIENT INFORMATION

(If female) Is the patient pregnant? ☐ YES ☐ NO ☐ UNKNOWN

If YES, **How far along?** ______________________

Is the patient a healthcare worker (anyone involved with patient e.g. nurse, hospital cleaner, ambulance driver)? ☐ YES ☐ NO ☐ UNKNOWN

If YES, **Position**______________________ **Name of facility**__________________________

Location of facility: District _______________ Town/Village _________________

If NO, **Specify occupation**__________________________

Did the patient visit another health centre or traditional healerfor this illness (including pharmacy)?

☐ YES ☐ NO ☐ UNKNOWN

If YES, **Name of facility** ____________________ **District**___________________________

Date visited other facility (DD/MM/YYYY) [__][__]/[__][__]/2014 ☐UNKNOWN

Patient ID # in other facility ______________________________

Location where patient became ill: District__________________________________

Village_____________________ Chiefdom _____________________________

ADDITIONAL MEDICAL INFORMATION

Additional medical problems, chronic conditions, and current medications?

☐Yes ☐No ☐Unknown

Describe:

Patient ID _______________ Patient Name: ____________________

Has the patient had contact with someone with FHF or someone who has been ill recently?					
Name of FHF contact	Relationship	Date of contact	Type of contact*	Phone Number	Village/Chiefdom

*In case of contact with someone with FHF (or probable FHF), what was the closest contact:

1 - Slept in same house within the last 21 days.
2 - Had direct physical contact.
3 - Touched their body fluids (excreta, vomit etc.)
4 - Had sexual relations.
5 - Handled clothes or other personal objects.
6 - Suckled patient or breast-fed from patient.
7 – Contact with body at a funeral
8 – Contact with the mattress, clothing or coffin of body during funeral practices.
(Not including contact with rope for lowering the coffin nor touching earth during burial.)

DRUG REQUEST FORM – IMC – LUNSAR EBOLA TREATMENT CENTRE

DATE: [___][___]/[___][___]/ 20__ (DD/MM/YYYY)	PATIENT ID #: LU– [__] – [__][__][__][__][__] LU-2 = Triage; LU-3 = Confirmed; LU-4 = morgue

BASIC PATIENT INFORMATION

Ward #: ______ **Bed #:**______

Name: Surname______________________ Given names______________________

Sex: ☐Male ☐Female **Age** [_____] ☐ years or ☐ months (for children < 1 y)

Is patient pregnant? ☐ YES or ☐ NO/UNKNOWN

If YES, **which trimester**: ☐first ☐second ☐third

PRESCRIBER INFORMATION

Name of prescriber: ______________________ **Prescriber ID #:** ______________________

MEDICATION

Antimalarials

- ☐ **Artemether-Lumefantrine (ACT)**
- ☐ **Artusunate**
- ☐ **Artemether**

Antibiotics

- ☐ **Ceftriaxone**
- ☐ **Cefixime**
- ☐ **Metronidazole**

Analgesics/Antipyretics

- ☐ **Paracetamol**
- ☐ **Tramadol**
- ☐ **Morphine**

Other

- ☐ **Vitamin A**
- ☐ **Vitamin C**
- ☐ **Zinc sulphate**
- ☐ **Diazepam**
- ☐ **Ondansetron**
- ☐ **Haloperidol**
- ☐ **Chlorpromazine**
- ☐ **Ivermectin**
- ☐ **Omeprazole**
- ☐ **Ranitidine**
- ☐ **Other (list)**__________________

LAB REQUEST FORM – IMC – LUNSAR EBOLA TREATMENT CENTRE

DATE: ____ / ____ / 20__
DD / MM / YYYY

PATIENT ID #: LU– ☐ – ☐☐☐☐☐
LU-2 = Triage; LU-3 = Confirmed; LU-4 = morgue

BASIC PATIENT INFORMATION

Ward #: ______ **Bed #:**______

Name: Surname______________________ Given names______________________

Sex: ☐Male ☐Female

Age: _____ ☐ YEARS or ☐ MONTHS (for children under 1 year)

PRESCRIBER INFORMATION

Name of clinician: ________________________ Clinician Signature: ____________________

Position: ☐Doctor in charge ☐Head nurse ☐Community Health Officer

BLOOD DRAW INFORMATION

Blood Samples Drawn by:_________________________ Signature: __________________

Date blood Drawn: ____ / ____ / 20__ DD / MM / YYYY

Time Blood Drawn: ____ : ____ : ____ Hr : Min : AM/PM

LAB TESTS (PHE)

☐ **New admission (Malaria RDT + Ebola PCR)**

☐ **Repeat Ebola PCR**

LAB TESTS (MoD)

☐ **FBC/CBC**

☐ **Urea and electrolytes (U+Es)**

[Urea; Creatinine; Sodium; Potassium]

☐ **Liver function tests**

[bilirubin – conjugated/unconjugated; AST, ALT]

☐ **Albumin**

☐ **Calcium**

☐ **Amylase**

☐ **Glucose**

☐ **Lactate**

☐ **Clotting screen**

☐ **Group and X-Match**

☐**Urinalysis**

☐ **Blood culture**

☐ **Store Serum**

☐ **Other (specify): ___________________**

Provider Name:

Date/Time:

		Patient Names				
Vital Signs	Temperature °C					
	Heart rate / pulse					
	Respiratory rate					
	Consciousness	A V P U	A V P U	A V P U	A V P U	A V P U
Signs & Symptoms	Urine frequency					
	Stool frequency					
	1. Did patient eat?					
	2. Did patient drink?					
	3. Headache					
	4. Bone/muscle pain					
	5. Stomach pain					
	6. Weakness					
	7. Anorexia					
	8. Swallowing problems					
	9. Nausea					
	10. Vomiting					
	11. Diarrhoea					
	12. Breathlessness					
	13. Red/injected eyes					
	14. Non-hemorrhagic rash					
	15. Hiccups					

Provider Name:

Date/Time:

		Patient Names				
Signs & Symptoms (Cont'd)	16. Bleeding	None<br>Nose/Oral<br>Cough<br>Vomit<br>Stool<br>Vaginal (non-menstrual)<br>Other	None<br>Nose/Oral<br>Cough<br>Vomit<br>Stool<br>Vaginal (non-menstrual)<br>Other	None<br>Nose/Oral<br>Cough<br>Vomit<br>Stool<br>Vaginal (non-menstrual)<br>Other	None<br>Nose/Oral<br>Cough<br>Vomit<br>Stool<br>Vaginal (non-menstrual)<br>Other	None<br>Nose/Oral<br>Cough<br>Vomit<br>Stool<br>Vaginal (non-menstrual)<br>Other
	17. Other (describe)					
Clinical Notes	Notes					
Treatment	Treatment<br><br>(Write in any additional medications)	Standing Orders	Standing Orders	Standing Orders	Standing Orders	Standing Orders

Nurse Inpatient Rounding Form **Provider Name** **Date/Time:**

Ward/ Bed #	Name / ID	Temp °C	Heart / Pulse rate	Respiratory Rate	Nurses Notes / Patient Requests

Weight: _________kg (children only)

Treatment: Doctors write in dosage and route under medication name (see formulary), then place circles for when to give medication. Nurses mark an X through the circle when medication is given.

Date	_____/_____			_____/_____			_____/_____			_/_____		
Shift	AM	AFT	NGT	AM	AFT	NGT	AM	AFT	NGT	AM	AFT	NGT
Day	1	1	1	2	2	2	3	3	3	4	4	4
ORS	O	O	O	O	O	O	O	O	O	O	O	O
Cefixime	O			O			O			O		
Coartem	O		O	O		O	O		O			
Paracetamol	O	O	O	O	O	O	O	O	O	O	O	O
Omeprazole	O			O			O			O		
Vitamin A	O			O								
Vitamin C	O	O	O	O	O	O	O	O	O	O	O	O
**Multi-Vitamins**												
Extra ORS												
Ringers Lactate												
Morphine sulfate												
Morphine HCL												
Metoclopromide												
Zinc Sulfate												
Diazepam												
Lorazepam												
Chlorpromazine												
Halperidol												
Ceftriaxone												
Ciprofloxacin												

Weight: _________kg (children only)

**Treatment: Doctors write in dosage and route under medication name (see formulary), then place circles for when to give medication. Nurses mark an X through the circle when medication is given.**												
Date	_____/_____			_____/_____			_____/_____			_/_____		
Shift	AM	AFT	NGT	AM	AFT	NGT	AM	AFT	NGT	AM	AFT	NGT
Day	1	1	1	2	2	2	3	3	3	4	4	4
ORS												
Cefixime												
Coartem												
Paracetamol												
Omeprazole												
Vitamin A												
Vitamin C												
**Multi-Vitamins**												
Extra ORS												
Ringers Lactate												
Morphine sulfate												
Morphine HCL												
Metoclopromide												
Zinc Sulfate												
Diazepam												
Lorazepam												
Chlorpromazine												
Halperidol												
Ceftriaxone												
Ciprofloxacin												

DISCHARGE FORM – IMC – LUNSAR EBOLA TREATMENT CENTRE

DATE: ____ / ____ / 20__
DD / MM / YYYY

PATIENT ID #: LU– ☐ – ☐☐☐☐☐
LU-2 = Triage; LU-3 = Confirmed; LU-4 = morgue

COMPLETE FORM UPON DEATH OF PATIENT, DISCHARGE, or TRANSFER

Final outcome: ☐ Deceased ☐ Discharged ☐ Transferred to other facility

***If Deceased*, date of death:** _____ /_____ / 20_____
DD / MM / YYYY

date transferred to morgue: _____ /_____ / 20___
DD / MM / YYYY

date of burial: _____ /_____ / 20_____
DD / MM / YYYY

If Discharged,

Discharge type: ☐ By staff ☐ Self-discharged ☐ Removed by family ☐Unknown

Did the patient have a confirmed negative test for Ebola? ☐YES ☐NO

If yes, ☐ never had Ebola (discharged from suspect ward)
OR
☐ recovered from Ebola (discharged from recovery ward)

Discharge medications provided?

If yes, list medications ____

If Transferred to other facility,

Reason for transfer: ____

Name of new facility: ____

District/town of new facility: ____

Discharge medications provided?

If yes, list medications ____

☐ Refer to psychosocial Team upon completion

Form completed by (print name): ______________________________________

Signature: ____
