# Supplementary material for: The natural history of acute Ebola Virus Disease among patients managed in five Ebola treatment units in West Africa: A retrospective cohort study
Source: PLoS Negl Trop Dis. 2017 Jul 19;11(7):e0005700. doi: 10.1371/journal.pntd.0005700 (PMC5536498; doi:10.1371/journal.pntd.0005700)
Supplement: S2 Appendix — (PDF) [file pntd.0005700.s002.pdf]

Viral Hemorrhagic Fever Draft Guidelines
Standard Clinical/Psychosocial Procedures for Ebola Treatment Unit (ETU) Operations*

*This document provides a general overview of procedures, which varied slightly by ETU and over time.

Table of Contents

ETU Triage Algorithm for Suspect Cases ……………………………………………………………….3

Standard Clinical Procedures ………………………………………………………...…………………4

Admission ………………………………………………………………………...………………….… 4

Daily Rounds ……………………………………………………………………………….....4

Laboratory Testing …………………………………………………………………….....5

Adult Clinical Management (>15 years of age)………………………………………………… ………6

Standing Orders: Medications for Adults …………………………………………..……….6

Symptomatic Care Guidelines and Medications ……………………………...…………….6

Assessment and Management of Dehydration……………………………..…….……….. 9

Discharge Medications ………………………………………………………………….10

Pediatric Clinical Management Guidelines (<15 years of age)………………………………………... 11

Standing Orders: Medications for Children…………………………...…………….. 11

Symptomatic Care Guidelines and Medications …………………………………………..11

Assessment and Management of Dehydration …………………………………………..…14

Special Considerations for Children ……………………………………..…………..15

Discharge Medication …………………………………………………………………..15

Clinical Management Guidelines for Pregnant Women……………………………………..……… 16

Pregnancy Testing on Admission …………………………………...………………..16

Key Messages for Patient Counselling……………………………………………………….. 16

Principles of Management for Pregnant Women with Ebola ……………………..………..16

Labor and Delivery Procedures …………………………………………………......….17

Management of Obstetric Problems ……………………………………………………….19

Waste Management………………………………………………………………….. 20

Newborn/Infant Feeding……………………………………………………………….….. 20

Discharge of Pregnant/Post-Delivery/Post Miscarriage Women ..……………… ………..…23

Psychosocial Support ……………………………………………………………………………..23

Admissions Protocol- Initial Contact …………………………………………………...24

Follow up- Second Contact ……………………………………………………………..24

Continuing Care: Follow up during ETC Stay ……………………………………… ….…25

Annex I: Laboratory/IV Procedure………………………………………………………......26

Annex II: Management of Malaria in an ETU………………………………………………………..28

Annex III: Pediatric Fluids Guidelines…………………………..…………………………………...30

ETU Triage Algorithm for Suspect Cases

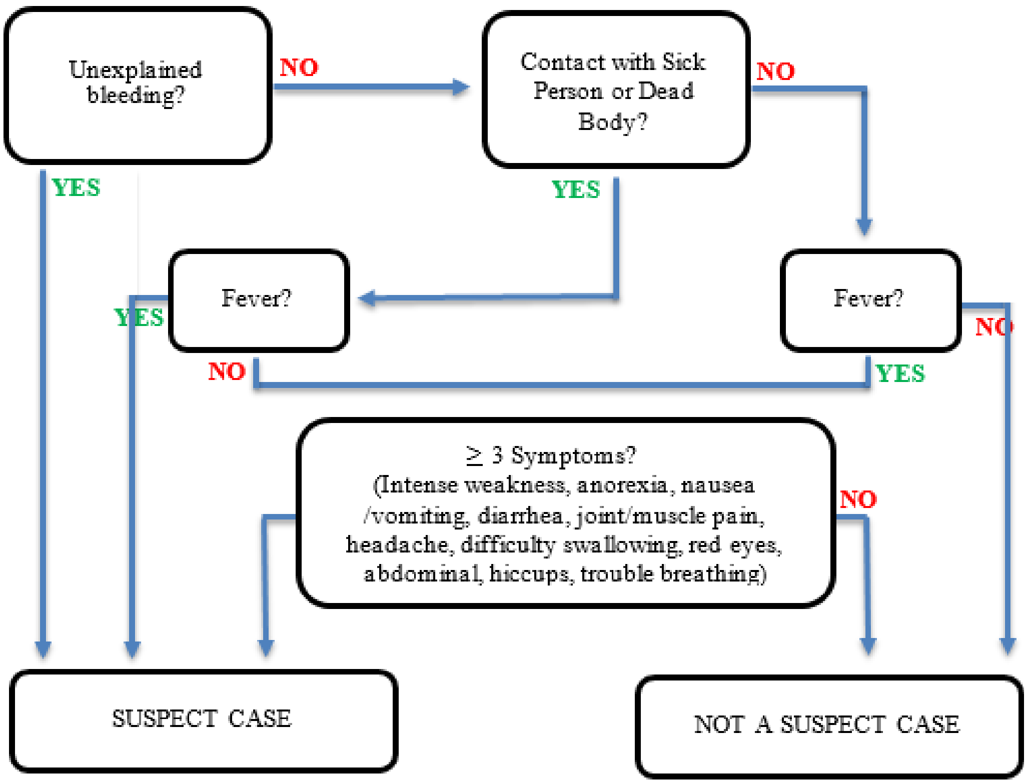

Standard Clinical Procedures

Admission

1. An admission form/epidemiological tracing form will be filled out by the triage nurse for all patients on arrival:
    a. Patient meets the triage criteria for a suspect case (see triage criteria) and will be admitted to the suspect ward
    b. Patient had a positive test for Ebola in the community or another facility and will be admitted to the confirmed ward
    c. Patient is dead on arrival to the ETU but meets the triage criteria for a suspect case or had a positive Ebola test in the community or at another facility and will be moved to the morgue

2. After filling in the admission/epidemiologic form, the patient's name and ID should be written on a bracelet by the triage nurse.

3. Next the triage nurse should fill in a lab slip for the patient and write their name and ID on an EDTA tube.

4. The triage nurse should explain to the patient that they are being admitted to the ETU and what to expect once they are admitted.

5. Two hygienists in PPE should place the bracelet on the patient's wrist and then escort (or carry) the patient to the suspect (or confirmed) ward. The triage nurse should hand over the bracelet, lab slip, and EDTA tube for the hygienists to take with the patient.

6. Afterwards, disinfection of the area where the patient sat or came into contact with should be sprayed with 0.5% chlorine solution.

Daily Rounds

1. A physician/physician assistant will round on all patients in the ETU at least once a day. During their rounds they will:
    a. Assess the patient clinically
    b. Fill in the daily symptom log
    c. Write orders for medications/labs as needed

2. Nurses will round on all patients at least 4 times per day (morning, mid-day, evening, and night). During rounds they will:
    a. Check weight (for children) on their first day
    b. Check patient temperature, pulse, and respirations once per day
    c. Give patients oral and intravenous medications and fluids
    d. Perform lab testing as ordered

3. Nurse assistants will round on all patients at least 4 times per day (morning, mid-day, evening, and night). During rounds they will:
    a. Deliver food to patients and encourage them to eat
    b. Give water/ORS to patients and encourage them to drink
    c. Clean and help change patients as needed

4. Psychosocial (PSS) will round on all patients at least once per day. During rounds they will:
    a. Check on patients well-being, comfort and needs
    b. Provide basic PSS support in line with principles of PFA
    c. Ensure connections and communication with family

Laboratory Testing

1. All patients should have an intravenous catheter placed and their blood drawn for Ebola testing on arrival in an EDTA (purple top) tube. See Annex 1 for Laboratory/IV Procedure.
2. Initial testing should also include PCR for malaria.
3. Result of initial lab test will determine whether the patient remains in suspect/probable ward or moved to confirmed ward.
4. If initial test negative patient will remain in ETU and have further PCR test to ensure 2 negative tests prior to discharge.
5. Patients who receive initial positive test will be moved to the confirmed ward, further PCR testing will only take place after patient becomes symptom free.
6. All patients who die during admission to the suspect and probable ward or who are dead upon arrival to the facility will have a buccal swab taken for PCR testing prior to being moved to the morgue.

Adult Clinical Management Guidelines (≥ 15 years of age)

Standing Orders: Medications for Adults

Existing Medical conditions – Every effort should be made to keep patients on essential medications for existing medical problems, including HIV & TB

1. Oral Rehydration Solution (ORS) 1.5 liters per day by mouth
2. Cefixime 400mg tab PO once a day for 5 days
   a. Alternatively, ciprofloxacin 500mg twice daily for 5 days for patients >7 years
3. Artemether/Lumefantrine (Coartem) 4 tabs PO twice a day for 3 days
4. Paracetamol 1 g (2 tabs) PO up to 4 times a day (as needed for pain/fever)
5. Omeprazole 20 mg PO once a day
6. Vitamin A 200,000 IU (2 tabs) by mouth once daily on days 1, 2, and 8
7. Vitamin C 500mg (2 tabs) by mouth 3 times per day
8. Multivitamin: 1 tablet daily

Symptomatic Care Guidelines and Medications (not all medications may be available/on formulary)

Diarrhea

1. Loperamide 4 mg x1, followed by 2 mg/dose after each stool up to a max dose of 16mg/24hrs
2. Many of the patients who die of EVD actually die from dehydration due to diarrhea. We can prevent these deaths by rehydrating patients appropriately.
3. All patients with diarrhea should be assessed for signs of dehydration
   a. (See Assessment and Management of Dehydration for Adults and Children page 9 and 15)

Hiccups

Chlorpromazine 25 mg tab twice a day

Suspected Hypoglycemia (and unable to take PO nutrition): Start IV line if feasible Give Dextrose 10% 250cc rapid infusion

Severe Malaria: REFER TO ANNEX I

1. Artemether: IM

Give a loading dose of 3.2mg/kg (maximum 160mg), followed by 1.6mg/kg (80mg) for the following 4 days.
Give a minimum of 2 doses, then switch to oral treatment (Coartem 4 tablets BID x 3 days) as soon as the patient is able to tolerate oral medications.

2. Artesunate IV/IM (refer to Malaria management guide for preparation instructions and dosing chart.)

Give 2.4 mg/kg every 12 hours for the first three doses (time 0, 12 hr and 24 hr), then give 2.4 mg/kg dose once daily for maximum of 6 days. Use a minimum of 3 doses before switching to oral Coartem 4 Tablets twice daily for 3 days.

Pain control: ** Use one medication option at a time. Do NOT use NSAIDs (ibrufen, aspirin, or diclofenac due to risk of bleeding)

1. Pain is a common symptom of Ebola Virus Disease (EVD), including headache, sore throat, neck pain, back pain, chest pain, abdominal pain, joint pain, and muscle pain.

2. Treating pain of both adults and children is an important part of care for patients in the ETU.

3. Patients who are unable to swallow tablets can receive morphine by IV:
   a. Morphine hydrochloride 0.1mg/kg IV four times per day as needed for both adults and children

b. If the patient does not have an IV in place, then morphine hydrochloride can be given sub-cutaneous (SC) at the same dosage
c. Avoid intramuscular (IM) injection due to risk of bleeding into muscle and formation of hematoma

Pain level	Medication	Dosing
Mild pain	Paracetamol	1g PO 6h PRN, maximum 4g/24 hours
Moderate pain	Tramadol	50 mg – 100 mg PO up to 4 times a day
Severe pain	Morphine	2-20 mg PO/IV up to 4 times a day (sustained release tab only twice a day)

Nausea or Vomiting: Oral medications preferred unless unable to tolerate

1. We should treat nausea early in order to prevent patients from vomiting and also to help them eat and drink.

	Medication	Dosing
** Use one medication option at a time. Treat early to maximize success of ORS	Ondansetron	4-8 mg PO/IV up to 4 times a day
	Promethazine	25 mg PO up to 4 times a day
	Metoclopramide	10 mg PO/IV up to 4 times a day

Anxiety

1. Many patients with Ebola will have anxiety or confusion at some point during their stay in the ETU. It is important to treat symptoms of anxiety or confusion **early** before they become more severe and difficult to manage.
2. If patients are experiencing anxiety or confusion, they should be provided basic PSS support including listening supportively to their concerns, providing reassurance and meeting unmet needs. The PSS team should also be consulted and can provide additional support.
3. Patients with moderate to severe anxiety who are not sufficiently responding to basic PSS support and are able to swallow pills should be given diazepam:
4. Patients with moderate to severe confusion or agitation who are not sufficiently responding to basic PSS support can be given chlorpromazine:
5. Patients with severe confusion or agitation should be treated with an IV medicine if they have an IV in place already and it can be done safely:
    a. Adults: haloperidol 5mg IV or lorazepam 1mg IV as needed
6. **You should never try to place a new IV or give an injection in a patient with confusion or agitation.**
7. If absolutely needed for a patient's safety, soft restraints (i.e. bed sheets) can be used to tie a patient to their bed to keep them from falling off.
    a. If soft restraints are being placed, at least two limbs should be restrained (1 arm and 1 leg, or 2 arms, or 2 legs).
    b. Patients need to be monitored consistently while restrained
    c. The restraints should be removed as soon as it is safe to do so.
    d. PSS support should be provided before, during and after an episode of severe confusion or agitation

	Medication	Dosing
** Before medication options consider reassurance and psychosocial support across visitors fence if patient can sit outside	Diazepam	2.5 mg – 5 mg PO/IV up to 4 times a day
	Haloperidol	2.5 mg – 5 mg IM up to 4 times a day

Confusion/Agitation

** Treat early. Patient and staff safety critical. DO NOT start IVs in confused or agitated patients<br><br>** Use one medication option at a time.	Medication	Dosing
	Chlorpromazine	50 mg PO up to 3 times a day
	Haloperidol	2.5 mg – 5 mg IM up to 4 times a day
	Diazepam	5 mg – 10 mg PO/IV up to 4 times a day

Seizures/Convulsions

Prepare 2 doses (loading dose and repeat dose) and bring into the unit at the same time.

Medication	Dosing
Diazepam	IV: 5 mg, if still seizing after 5 minutes, repeat 5 mg. If still seizing, consider additional pathology (meningitis, hypoglycemia…)
	Rectal: 10 mg, if still seizing after 5 minutes, repeat 10 mg. If still seizing, consider additional pathology (meningitis, hypoglycemia…)
Phenobarbital	• 15mg/kg loading dose IM x 1<br>• If still having seizures after 15-20 minutes give a 10mg/kg IM dose x 1<br>• If seizures stop, start maintenance dose of 5mg/kg IM once daily.<br>• If continue seizures, consider additional pathology (hypoglycemia, meningitis) and treat appropriately

Hemorrhage

1. Many patients with Ebola will have some form of bleeding. The most common sites of bleeding will be blood in the stool, bleeding from the vagina, bleeding from the gums, bleeding from the nose, vomiting blood, and bleeding from an IV catheter site.
2. For bleeding from an IV catheter site:
    a. If the IV is no longer needed, the IV should be removed and the site should be bandaged well to stop bleeding.
    b. If the IV is still needed and there is only a little bleeding, wrap additional bandage around the arm to stop the bleeding.
    c. If there is a lot of bleeding from the catheter site, remove the IV and bandage the site well to stop bleeding.
3. For bleeding from the nose, instruct the patient to pinch just below the bridge of their nose tightly for 30 minutes at least.
4. For bleeding from the gums, instruct the patient to rinse their mouth with clean water several times a day and spit out into their bucket.
5. For vomiting blood, give metoclopramide as instructed in the nausea/vomiting section above.
6. For blood in the stool, double the patient's current dose of omeprazole from 20mg to 40mg for an adult

Other

Allergic Reaction: Dexamethasone 4 mg tabs, Give maximum 12 mg (3 tabs) PO x 1

Electrolyte Abnormalities

1. Potassium: 2 tabs of 600 mg or 750 mg orally once daily until diarrhea resolves (empiric treatment)

    a. Patients in whom formal potassium levels unavailable

        - All patients should receive ORS, which contains around 20 mEq/L of potassium.

- Patients with ANY vomiting or diarrhea and tolerating oral intake, prescribe 40mEq per day of oral potassium replacement in addition to ORS.
- If large volume diarrheal loss or vomiting, empirically add 20mEq of potassium to each liter of IV fluid given.
- If oral intake possible, continue giving 40mEq oral potassium per day IN ADDITION TO IV replacement.
- If patient is oliguric/anuric and formal laboratory results unavailable, use caution with potassium replacement and discuss with senior clinical team.
- If requiring IV maintenance and no vomiting or diarrhea, give maintenance potassium in Hartman’s/Ringer’s/saline at 20mmol IV per day.

b. Patients with formal laboratory potassium levels available

- As per admission potassium levels

2. Magnesium: 2g Magnesium in 1L of 5% dextrose or normal saline infused over 45-60 minutes. Do not use pressure bag.

- Once per day for 3 days.
- If large volume watery stools ongoing, repeat again after 72 hours.

Assessment and Management of Dehydration

Any decision to start an IV line to give IV medication or IV fluids must take into consideration staff and patient safety. It may be safest to avoid IV treatment in patients who are confused or disoriented and cannot be appropriately monitored.

Giving ORS does not mean just putting the bottle of ORS by the patient!

1. Adult patients should be encouraged to drink small sips every few minutes until the bottle is finished. Dehydrated patients may need supervision to take adequate ORS
2. Patients who are too weak to lift the bottle should be given a straw made from a piece of IV tubing to drink the ORS.

Dehydration Classification & Management

Dehydration Stage	Signs	Treatment
**Severe / shock With No malnutrition**	Lethargic, unconscious, floppy Very sunken eyes, drinks poorly or is unable, mouth very dry Skin pinch goes back very slowly Weak or absent pulse<br>Capillary refill > 3 sec Tachycardia out of proportion to fever	•Lactated Ringers (LR) 2 liters IV infusion over 30 minutes<br>•If patient continues to show signs of severe dehydration, Repeat LR 2 liters IV infusion over 30 minutes as soon as possible<br>•Continue this process until the Patient is no longer showing signs of dehydration/shock<br>•Encourage ORS as soon as patient is able
**Severe/shock with SEVERE malnutrition (if the patient looks wasted/cachetic)**	Lethargic, unconscious, floppy Very sunken eyes, drinks poorly or is unable, mouth very dry<br>Weak or absent pulse<br>Capillary refill > 3 sec Tachycardia out of proportion to fever	•Lactated Ringers (LR) 1 liter IV infusion over 30 minutes<br>•If patient continues to show signs of severe dehydration, Repeat LR 1 liters IV infusion over 30 minutes as soon as possible<br>•Continue this process until the patient is no longer showing signs of dehydration/shock<br>•Encourage ORS as soon as patient is able
**Moderate**	Restless and irritable, sunken eyes<br>Dry mouth, thirsty, drinks eagerly Skin pinch goes back very slowly	•Encourage 3 liters ORS over 4 hours until hydration status improved to mild or none<br>•Consider NG tube if no signs of bleeding or IV for anyone unable to take in at least standard dose of ORS

**Mild / No Dehydration**	Thirsty, drinks eagerly None of the above signs	ORS per standing orders (1.5 liters per day)

Discharge Medications

If Adult patient is PCR negative for Ebola and discharged from ETU but febrile or ill, examine fully outside ETU

Diarrhea (bloody), Upper Urinary Tract Infection: Ciprofloxacin: 500 mg PO 2 times per day for 7 days

Skin infections: Clindamycin: 600 mg PO 3 times per day for 7 days

Amoebiaisis, Giardiasis: Metronidazole: 500 mg PO 3 times per day for 7 days

Pediatric Clinical Management Guidelines (< 15 years of age)

Existing Medical conditions – Every effort should be made to keep patients on essential medications for existing medical problems, including HIV & TB

Standing Orders: Medications for Children

1. **Oral Rehydration Solution (ORS)**
   a. 40ml/kg per day of ORS by mouth with no or mild dehydration 80ml/kg per day with moderate dehydration
   b. Can be given by NG tube if not vomiting, no evidence of bleeding and unable to take sufficient quantity by mouth
   c. Refer to oral fluid dosing chart for appropriate volumes to give per shift.
2. **Cefixime** 8mg/kg solution by mouth once daily for 5 days, if able to tolerate oral medications
3. **Ceftriaxone** (if unable to tolerate oral antibiotics): 75-100mg/kg once daily x 7 day
4. **Artemether/Lumefantrine (Coartem)** (uncomplicated malaria): If able to take oral medications. Refer to Malaria Treatment Guidelines for management of severe malaria.

Weight	Dosing
5 to 14.9 kg	1 tab. initially, 1 tab. at 8 hrs and then 1 tab. BID x 2 days (total 6 tab.)
15 to 24.9 kg	2 tab. initially, 2 tab. at 8 hrs and then 2 tab. BID x 2 days (total 12 tab.)
25 kg to 34.9kg	3 tab. initially, 3 tab. at 8 hrs and then 3 tab. BID x 2 days (total 18 tab.)
Greater than 35 kg	4 tab. initially, 4 tab. at 8 hrs and then 4 tab. BID x 2 days (total 24 tab.)

Coartem can be crushed and mixed in water for small children

5. **Paracetamol:** 15mg/kg by mouth 4 times per day (as needed for pain/fever
6. **Omeprazole:**
   - **< 20 kg: 10 mg, ≥ 20 kg: 20 mg**
     Note: can dilute ½ capsule powder in small amount of ORS/RUIF and give orally with syringe
7. **Vitamin A: Age 6-12 months: 100,000 IU; Age > 12 months: 200,000 IU**
8. **Vitamin C:** 250mg by mouth 3 times per day (crush and mix in water)
9. **Multivitamin:** 1 tablet daily

Symptomatic Care Guidelines and Medications (not all medications may be available/on formulary)

Suspected Hypoglycemia (and unable to take PO nutrition)
Start IV line if feasible and give D10 5ml/kg rapid infusion, repeat as needed at next clinical encounter

To prepare 10% glucose solution:
Add 10ml of 50% glucose to 90ml of 5% glucose solution OR 10 ml of 50% glucose solution to 40ml of sterile water.

Pain (for moderate to severe pain not controlled by Paracetamol)
** Use one medication option at a time. Do NOT use NSAIDs.

1. Tramadol
   FOR CHILDREN > 25 kg (or 15yrs age) - 1-2 mg/kg PO up to 4 times a day (maximum dose is lesser of: 8mg/kg/day or 400 mg/day)
2. Morphine: IV/INTRANASAL DOSING 0.05-0.1 mg/kg/dose IV up to 4 times a day (max dose 15 mg/dose for children); REFER TO DOSING CHART FOR APPROPRIATE WEIGHT-BASED DOSING. Must administer IV dose followed by 5 ml flush with Ringers Lactate.

3. Morphine: ORAL DOSING - 0.2-0.4 mg/kg (max dose 15 mg/dose for children); REFER TO DOSING CHART FOR APPROPRIATE WEIGHT-BASED DOSING.

Nausea or Vomiting
** Use one medication option at a time. Treat early to maximize success of ORS

1. Ondansetron: 0.2mg/kg, Oral or IV, up to 4 times a day, children < 4 years: 2mg, ≥ 4 years: 4 mg
2. Promethazine: *FOR CHILDREN AGED 2 yrs or older*: (0.25-1mg/kg) Oral, up to 4 times a day
3. Metoclopramide for Children > 10 kg; 0.1-0.2 mg/kg PO/IV up to 4 times a day

Anxiety
*** Before medication options, consider reassurance and psychosocial support across visitors fence if patient can sit outside, Refer to dosing chart for weight based doses*

1. **Diazepam:** 0.1mg/kg PO/IV up to 4 times a day, (maximum dose 0.6mg/kg within 8 hour period)
2. **Lorazepam** 0.05 mg/kg/dose up to 4 times per day, **maximum single dose: 2mg**

Confusion/Agitation
** Treat early. Patient and staff safety is critical. Be very cautious starting IVs in confused or agitated patients.
**REFER TO DOSING CHARTS FOR WEIGHT BASED DOSES.

1. Diazepam: 1.1 mg-0.2mg/kg PO/IV up to 4 times a day (maximum dose 0.6 mg/kg within an 8 hour period)
2. **Lorazepam** 0.05 mg/kg/dose up to 4 times per day, **maximum single dose: 2mg Haloperidol:**
    a. Younger than 6 years: DO NOT USE
    b. 6 years to 12 years: 1-3mg/dose up to 4 times per day, max dose 0.15 mg/kg/24 hr
    c. > 12 years: 2-5 mg/dose, repeat in 1 hour as needed for agitation, up to 4 times per day

Diarrhea (only for children under 5 years old) Offer
Bananas to replenish potassium losses

1. Zinc:
    a. ≥ 6 months – 5 years: 20 mg tab once per day for 10 days (can mix with water/ORS)
    b. < 6 months: Zinc Sulfate 10 mg tab once per day for 10 days (can mix with water/ORS)
2. Loperamide

Age	Dose
less than 2 years	DO NOT USE
2-5 years	1 mg orally, 3 times daily
6-8 years	2 mg orally, 2 times daily
9-12 years	1 mg orally, 3 times daily

Note: Max single dose: 2 mg

Hemorrhage
Bleeding from an IV catheter site:
1. Remove IV if it is no longer needed – bandage the site well to stop bleeding
2. If the IV is still needed:
    a. Wrap additional bandage around the arm to control the bleeding
    b. If bleeding cannot be controlled, remove the IV and bandage the site
3. Bleeding from the nose - instruct the patient to pinch just below the bridge of their nose (or have the caregiver pinch the nose) tightly for 30 minutes at least.
4. Bleeding from the gums - instruct the patient/caregiver to rinse their mouth with clean water several times a day and spit out into their bucket.
5. Vomiting blood, give metoclopramide as instructed in the nausea/vomiting section above.
6. Bleeding in the stool - double the patient’s current dose of omeprazole from 10mg to 20mg

Seizures/Convulsions

Prepare 2 doses (loading dose and repeat dose) and bring into the unit at the same time. **Diazepam IV/RECTAL (**5mg/ml): 0.2-0.5mg/kg/dose every 5-10 min if seizures continue. Max total dose in < 5 years is 5mg, maximum total dose in > 5 years is 10 mg.

Phenobarbital

1. Neonates (< 1 month of age) 20mg/kg IV or IM instead of diazepam. If convulsions continue, give 10mg/kg IV or IM after 30 minutes (max total of 30mg/kg)
2. Infants/Children > 1 month of age: 15mg/kg loading dose IM x 1
3. If still having seizures after 15-20 minutes give a 10mg/kg IM dose x1
4. If seizures stop, start maintenance dose of 5mg/kg IM once daily.
5. If continue seizures, consider additional pathology (hypoglycemia, meningitis) and treat appropriately

Severe Malaria: REFER TO ANNEX I

PREFERRED:

1. Artemether: IM loading dose: 3.2 mg/kg; then maintenance dose 1.6 mg/kg once daily. Give maintenance dose daily for a minimum of 3 days until patient able to take oral treatment (maximum 6 days treatment).

ALTERNATIVE:

2. Artesunate: Give 2.4mg/kg every 12 hours for the first three doses (time 0, 12 hr and 24 hr). Use a minimum of 3 doses before switching to oral Coartem. Give by bolus over 1 minute. If unable to tolerate oral medications continue 2.4mg/kg once daily for a total of 6 days. Change to oral treatment as soon as child able to take sips.

Others

Fluid Overload: Furosemide: 1-2 mg/kg IV twice daily (maximum pediatric dose 2 mg/kg/dose, maximum adult dose 80mg/kg/dose)

Severe Croup, Allergic Reaction, Asthma: Dexamethasone 0.6mg/kg PO single dose

Electrolyte abnormalities: Electrolyte abnormalities are common in EVD and are likely to be a significant contributing factor to, or cause of, death.
Concomitant hypomagnesaemia is common in hypokalaemia in EVD patients. Potassium replacement is ineffectual with hypomagnesaemia - See below for pediatric magnesium protocol for appropriate correction in large volume fluid loss.

1. POTASSIUM
   Children in whom formal potassium levels <u>unavailable:</u>
   - All children should receive ORS 40ml/kg/day without dehydration, 80ml/kg/day with moderate dehydration, as per the oral fluid management table
   - If a child has diarrhea or vomiting further replacement of potassium should be given:
     - If child can manage oral fluids, give one 600mg KCl tablet (8 mEq of potassium) crushed in water and added to ORS or infant formula, once daily, until diarrhea resolves.
     - If child needs IV fluid replacement, potassium should be added to maintenance fluid and run slowly over 12 hours. Add 5ml of KCl (10mEq of potassium) to 500ml of fluid once daily, until diarrhea and vomiting resolves or oral potassium replacement can be given.
   - If child is oliguric/anuric and formal laboratory results are unavailable, use caution with potassium replacement and discuss with senior clinical team.

   Children in whom formal potassium levels <u>available:</u> Follow the protocol above, but discuss with senior medical lead if K+ less than 3.0.

2. MAGNESIUM

If potassium level is low magnesium must also be replaced.

When and how much magnesium to add:

If child has a low potassium level (<3.5 if level available) **OR** severe diarrhea, defined as > 3 days of large volume diarrhea and requiring on-going IV fluids, then replace magnesium.

Magnesium should be added to maintenance fluid once in every 24 hours.

The dose needs to be calculated for individual children and should be 0.2mmol/kg, (0.2mmol = 0.1ml)

Therefore give 0.1ml/kg. DO NOT GIVE MAGNESIUM BY IV PUSH!

Magnesium should be given once per day for 3 days then stopped. If large volume watery stool ongoing, repeat again after 72 hours.

Magnesium should not be given to children who are oliguric/anuric.

Assessment and Management of Dehydration

Any decision to start an IV line to give IV medication or IV fluids must take into consideration staff and patient safety. It may be safest to avoid IV treatment in patients who are confused or disoriented and cannot be appropriately monitored.

Giving ORS does not mean just putting the bottle of ORS by the patient!

1. Children should have a caregiver who can give them small sips using the cap of the ORS bottle.
2. Patients who are too weak to lift the bottle should be given a straw made from a piece of IV tubing to drink the ORS.

Classification and Management of Children with Dehydration

Dehydration Stage	Signs	Treatment
Severe dehydration or shock and **no malnutrition**	• Lethargic, unconscious, floppy<br>• Very sunken eyes, drinks poorly or is unable, mouth very dry<br>• Skin pinch goes back very slowly<br>• No tears (children)<br>• Weak or absent pulse<br>• Capillary refill > 3 sec<br>• Tachycardia out of proportion to fever	**Fluid bolus** (route as available) of Ringers Lactate:<br>• 20ml/kg x 1 over 20 min; repeat x 1 if no improvement<br>**Commence maintenance** with D5% or D10% in ½ strength Ringers Lactate<br><br>Consider increase to 1.5 x maintenance if significant losses (diarrhea/vomiting)<br><br>Continue IVF/NG fluids until hydration targets reached and tolerating oral fluids.<br><br>**Always encourage ad lib with ORS**
Severe dehydration or shock and **severe malnutrition Z-score < -3 SD**	• Lethargic, unconscious, floppy<br>• Very sunken eyes, drinks poorly or is unable, mouth very dry,<br>• Weak or absent pulse<br>• Capillary refill > 3 sec<br>• Tachycardia out of proportion to fever	**Fluid bolus** (route as available) of 5% Dextrose in Ringers Lactate:<br>• 15 ml/kg over 1 hour<br>*If improvement*, repeat 15 ml/kg bolus over 1 hour at next assessment, then switch to oral/NG fluids with Resomal every 6 hours. After 12 hours, continue Resomal and introduce F75, per nutrition protocol and offer food as tolerated.<br><br>*If no improvement* after initial bolus, give maintenance IV fluids at 4ml/kg/hour
Moderate Dehydration	• Restless and irritable, sunken eyes<br>• Dry mouth, thirsty, drinks eagerly<br>• Skin pinch goes back very slowly<br>• No tears (children)	**Oral Therapy:**<br>• ORS 80ml/kg/day or consider NG tube therapy
Mild / No Dehydration	• Thirsty, drinks eagerly<br>• None of the above signs	**Oral Therapy:**<br>• ORS per standing orders: 40ml/kg/day

REFER TO ANNEX II for PEDIATRIC FLUID MANAGEMENT GUIDELINES

Special Considerations for Children

- Less is known about Ebola in newborns and infants and it is possible that they may be infectious before becoming symptomatic, so negative newborns and infants should be handled cautiously even with a negative test. Testing should be repeated.
- If the child is negative (and it may be prudent to carry out two tests two days apart), he/she can leave pediatric isolation and be followed closely as a high-risk contact if there is a survivor or family member who can care for the child safely at home, while discouraging wet-nursing
- Special management is needed for children under 5 who are high-risk contacts but not yet Ebola suspects. Children can test negative then rapidly become symptomatic with high viral load and rapid deterioration. One approach to care is having Ebola survivors caring for children in light PPE, with vigilance for decreased appetite or playing or any lethargy as suspect signs. A special pediatric place (such as an observations interim care center) is desirable with attention to good nutrition and care to reduce stress.
- If the child is asymptomatic and his/her mother is symptomatic, the child should be separated from the mother if there is a safe alternative place to care for the child. If the child becomes sick and both the mother and child test positive for Ebola or Marburg disease, then the child can be returned to the mother.

Infants and young children

- Providing 24-hour care and psychological support for babies and small children is difficult in full protective clothing.
- To minimize risk of transmission, the staff should provide the care as much as possible. It should be anticipated that there might be infected children whose parents have died. The care of these children will require more time, therefore sufficient staffing must be planned and organized.
- However, for the well-being of both children and parents, parents should be permitted to stay in the unit to care for their children.
- The number of people at risk of infection is increased, but it may be easier for the family to arrange care provision.
- It is difficult for parents to stay for long periods inside the Treatment Ward in protective clothing, be it full or alternative PPE. Therefore, the length of time spent inside the Ward should be limited and the parent encouraged to take frequent breaks.

Older children of admitted patients

- If a patient admitted for suspected Ebola has children who are verbal and asymptomatic, they should stay at home, and be recorded and followed up as a contact.

Discharge Medications

If Pediatric Patient is PCR negative for Ebola and discharged from ETU but febrile or ill, examine fully outside ETU

Otitis Media (ear pain) Pneumonia: Amoxicillin: Give orally 3 times daily for 10 days

Diarrhea (bloody), Upper Urinary Tract Infection: Ciprofloxacin: 10-15 mg/kg/dose by mouth 2 times per day for 5 days (maximum 500mg per dose)

Skin infections: Clindamycin: 3-10 mg/kg/dose by mouth 3 times per day (max adult dose 1.8 g/24 hr)

Amoebiaisis, Giardiasis: Metronidazole: 7.5 mg/kg by mouth 3 times per day for 7 days

Clinical Management Guidelines for Pregnant Women (MSF Guidelines)

When a pregnant woman catches Ebola, the fetus and amniotic fluids are flooded with the virus. The ripple effect of these dangerous deliveries could be more catastrophic than Ebola itself.

Pregnant patients with Ebola are at increased risk of complications: postpartum hemorrhage (PPH), fetal demise/stillbirth, and spontaneous abortion

Pregnancy Testing on Admission

Pregnancy testing at admission is recommended if any suspicion of pregnancy from clinical examination or history. It could even be done as a standard test at admission if capacity allows it. In sites where experimental drugs will be tested (planned for December 2014/January 2015), pregnancy testing for all women of reproductive age will be part of the protocol.

Key Messages for Patient Counseling

- Inform any pregnant woman that the risk of miscarriage or early labor as part of Ebola illness is probable and that loss of fetal life is highly likely;
- Inform at appropriate time in recovery (e.g. when mentally aware, and potential for cure is real) that when cured, termination of pregnancy or induction of labor will be offered;
- Explain the reason for the induction of labor: very likely the fetus will be dead and the viral load in the fetus, placenta and amniotic fluid will still be high even after cure of the mother, making a risk for infection to birth assistants when delivering at home or a health facility.
- Explain that it is recommended that the woman to stay near the ETC when she is not ready for labor induction until she agrees or intrauterine fetal demise (IUFD) is confirmed.
- It is important that women understand the risks and reality of being pregnant with Ebola and that consent is obtained for any procedure;
- If very sick women go into labor/miscarriage and birth, chances of survival are minimal and interventions to save maternal life should be few and non-invasive (e.g. if possible oral misoprostol with bleeding; antibiotics if able to do so safely).

Principles of Management for Pregnant Women with Ebola

The over-riding principle is always the safety of all staff who are working within the High Risk area In the event of pregnant woman needing delivery inside the Ebola Treatment Centre (ETC) preparation of a “Maternity Box” with all needed drugs/equipment is advised.
Identification of staff able to assist the woman during/after delivery should also be pre- arranged if possible. There will usually need to be a sprayer and 2 medical/nursing staff with the patient, and at least one staff member in low risk as stand-by.

The pregnant women is contagious both by normal means as well as through amniotic fluid and breast milk, both of which remain Ebola virus positive after the women herself has survived.

The following principles are advised:

1. Always assess safety to healthcare and other workers. Numbers of people working in direct contact with patients and in the high risk area should be kept to a minimum and only appropriately qualified/trained personnel should enter. Invasive procedures should be kept to a minimum.
2. Any pregnant women suspected of having Ebola should be isolated and handled in the same way as any other suspect/conformed patient. This should remain the case until proven to not have Ebola. Begin the same standard treatment as you would for any other patient admitted to ETC.
3. No fetal monitoring is necessary. Viability of fetus can be assessed by asking the patient if she continues to feel fetal movements.
4. In the event of a women with, or recently survived of, Ebola delivering it should only take place within a designated high risk area (with privacy). Intravenous access should be secured at the earliest time to avoid risk of sharps injury in an emergency situation or with an agitated patient.
   a. Once again, fetal monitoring is not needed.
   b. Spontaneous vaginal delivery should be anticipated.

c. Vaginal examinations should be minimal and artificial rupture of membranes avoided.
d. Avoid standing directly in front of patient during delivery of fetus or placenta (deliver side- on) to avoid body fluid splash.
e. Misoprostal (Cytotec) 600mcg PO should be given immediately **after** delivery to reduce risk of bleeding (can be left with patient inside high risk area with instructions when to take)
f. Do not give an episiotomy. If there is a vaginal tear do not suture as there is high risk of health worker infection if sharps injury were to occur
g. Surgical interventions (for example if obstructed labor) are contra-indicated in an Ebola context. Continue to support the patient, provide pain relief and seek advice from obstetrician/midwife if possible.
h. It is common for pregnant women presenting with Ebola to have an intra-uterine fetal death (IUFD), current advice is to not induce labor until the women has recovered from Ebola. If spontaneous labor begins follow as above.

5. Placenta and stillborn child must be disposed of in accordance with high risk material protocol.
6. Misoprostol should be considered as first-line treatment for induction of labor, termination of pregnancy and post-partum hemorrhage. Intravenous/intramuscular drugs to be given with caution and only if the healthcare worker is appropriately trained and feels safe to do so. Intra-uterine procedures should not be performed. Spontaneous miscarriage is a common presentation in women infected with Ebola virus. Caution should therefore be taken in the assessment of women presenting with bleeding in pregnancy and an Ebola contact history. Expectant or medical management is advised. Do not perform vacuum or surgical uterine evacuation.
7. In the unlikely event of a live birth the baby must be assumed to be Ebola positive and handled in accordance with full personal protective equipment and safety protocols. If the mother wishes she may breastfeed as the baby is assumed to be Ebola positive, it should be made clear that the baby is very likely to die.
8. Mothers who continue to lactate, even if surviving Ebola, must be trained in safe breast-pumping and disposal of milk. Breast milk continues to carry the Ebola virus, this continues for an unknown length of time. Refer to the guidance paper for infant feeding advice.
9. **All** surviving women should be counseled and given adequate family planning, nutritional support and ferrous sulphate/folic acid.

Labor and Delivery Procedures

1. **Planning and Organization- Delivery in the ETC**

a. **Delivery Space**

One side in the confirmed cases tent needs to be installed for the delivery. The patient will deliver in the bed; a screen will provide some privacy and will be sprayed with chlorine after use.

b. **Delivery Equipment**

The needed materials should be reduced to the absolute minimum; all material needs to be discarded (burned) after use with the only exception of material that can be disinfected with chlorine and sterilized (recipient available in ETC). There is no need of a standard delivery kit (instruments). This can be replaced by plastic cord clamps and one pair of disposable scissors. When disposable scissors are not available, a regular surgical scissor with blunt tips should be used and discarded after use. Disposable blades cannot be used. Disposable absorbent drapes with plastic on one side should be used instead of reusable.

Practical aspects:

- Make sure all supplies are ready: some can stay in the delivery area, others should be brought last minute (eg. Oxytocin, prepared in low risk zone)
- No sharps should be left in the delivery area.
- Prepare a Maternity box: organize equipment beforehand in plastic bags per action (e.g. Items for delivery, items for bladder catheterization, items for PPH management, items for sample collection).

c. **Delivery Team**

It is best to have a gynaecologist, doctor or nurse/midwife with experience in deliveries, but this might not

always be feasible. Since the normal timeframe for a healthcare worker to stay in an ETC is only 1 (½) hours, a good handover between teams should be given every shift. Besides the two people per shift in the ETC, preferably two extra persons should be made responsible for when the patient delivers.

Practical aspects:
Personal protective equipment (PPE): "3 plus" as always in an ETC is mandatory
Recommended/suggested HR set-up – 4 staff (two pairs of two)

- One person with experience in obstetrics to lead the management of the patient +
- One person to assist in the delivery
- One person to observe respect of safety rules: intervention and time limits, hygiene and IC procedures +
- One chlorine sprayer

And: One time keeper in low risk zone: at the time of birth one extra responsible for time in low risk zone who is able to communicate between those inside to those outside to ensure team members are replaced in timely manner and safely and supplies are brought or passed in as needed (requested by team leader).

A 2nd team of 4 should be identified for follow up care when needed.

2. **Labor and Delivery Procedures**

REMINDER: Wash your gloved hands between each and every procedure as recommended. When planning to perform procedures: organize an extra person to observe respect of IPC procedures. All health workers MUST wash their hands with 0.05% chlorine solution before wearing the gloves and putting on PPE.

PREPARATION: If an IV line is to be used or anticipated, put it early (before labor or in early labor) to avoid needle stick injury by putting an IV line in an agitated patient) When possible, avoid IM/IV routes if oral medications available) (e.g. misoprostol PO for the prevention of postpartum haemorrhage instead of oxytocin IM).

a. **Induction and Delivery**

- ✓ **Routine antibiotic prophylaxis and antimalarial treatment:** antibiotics from admission until five days post discharge; combined with antimalaria treatment. Provide ferrous sulphate/folic acid for every pregnant woman or multivitamins from the moment of admission up to 1 month post discharge.

- ✓ **No induction of labor if the patient is still having fever,** await spontaneous labor even if this takes several days. **Opt only for induction of labor if the Ebola viraemia is negative (usually after 10-12 days after entry in the ETC)**. This is both to protect the medical staff and the patient. If the Ebola viraemia is negative, the risk of coagulation disorders might be less for the patient, and this might increase chance of survival for the mother. Induction can be done with misoprostol as per protocol. Artificial rupture of membranes should not be performed. The delivery will still be in the ETC (even with a negative viraemia in the blood, the amniotic fluid and placenta still contain virus for an unknown period as will the foetus). Give hygienic pad if membranes ruptured.

- ✓ **In case if the baby is still alive in utero**: **offer the mother planned termination of pregnancy in the ETC**. PCR in amniotic fluid remains positive even when the mother is cured (duration unknown but more than 30 days after cure of the mother PCR of placenta and cord blood was still Ebola positive in a case in Guinea). No baby has ever been reported to have survived transplacental Ebola. Termination of pregnancy should happen in the ETC to avoid Ebola infection of the family, TBA or health care workers. In case the mother is very motivated to keep the pregnancy: explain that chances of survival of the baby are extremely low. The mother should not leave the surroundings of the ETC in case she has not delivered yet.

Labor Induction

i. Term pregnancy

Before starting the induction, make sure the patient is not yet in spontaneous labor: regular and painful contractions (3 contractions every 10 minutes is a well-established labor). When possible, ask advice for induction from a gynaecologist or midwife.

Misoprostol 200 µg tablet: 25 µg PO (dissolve one tablet in 200 ml of water and give 25 ml of this solution) every 2 hours until good contractions are obtained. Do not exceed 150 micrograms total dose. If 2 hourly is not realistic, the dosage can be increased to 50 µg PO every 4 hours. Do not give misoprostol when there is history of a previous cesarean section.

ii. Labour induction of preterm pregnancies (2nd and 3rd trimester):

Misoprostol orally, every 6 hours, until labour begins (max. 3 doses within 24 hours, to be repeated after 24 hours if no good contractions yet): 200 micrograms in the second trimester or 100 micrograms in the third trimester or 50 micrograms in the ninth month. Ask for expert advice if patient is grand multipara (P5 or more) or has a previous cesarean section scar.

In case of prior caesarean section or grand multiparity, given the increased risk of uterine rupture: The combination of mifepristone + misoprostol should be favoured over misoprostol alone to reduce the number of misoprostol doses required. Try to obtain advice from a midwife or gynecologist at a specialized EVD maternity center, if available.

b. **During Labor & Delivery:**

i. NO invasive procedures are allowed: no episiotomy, no vacuum, no destructive delivery as these are contraindicated for safety and protection of medical staffs.
ii. Limit the number of vaginal examination; no artificial rupture of membranes during labor;
iii. Administer routine prophylactic antibiotic, antimalarial treatment, ferrous sulfate and multivitamin;
iv. No fetal monitoring is required since that no actions will be taken if it is not normal or absent. Ask the mother if she feels fetal movements/leakage of amniotic fluid. If no fetal movements for several days, discuss with the mother the high likelihood that the baby has died.
v. Clamping and cutting the umbilical cord are not to be done if the baby is stillborn, but if the baby is born alive, the umbilical cord is clamped with 2 plastic cord clamps and cut with disposable scissors.

c. **After Delivery**

i. After the delivery of the placenta, Misoprostol PO 600µg - 3 tablets (preferred) should be given systematically to all women to prevent PPH. Alternatively, Oxytocin 10 IU IM x1 can be given, and can be repeated 2 – 3 hours later in case hemorrhage persists.
ii. Active management of 3rd stage of labour (abdominal uterine massage with a protective sheath on the abdomen and Misoprostol 600 µg orally after birth of the baby (first choice) or oxytocin IM or slow IV 10 IU (second choice) as prevention of postpartum haemorrhage should be done.
iii. No controlled cord traction, wait until placenta delivers spontaneously
iv. When delivering the placenta, stay at the side of the mother to prevent splashes on the care provider and cover the area as much as possible.
v. After the delivery of the placenta, both the corpse of the stillborn and the placenta can be treated as per protocol
vi. NO suturing of any tears, episiotomy and vaginal examination not authorized after delivery

Management of Common Obstetric Problems

1. **POSTPARTUM HEMORRHAGE (PPH):**

a. For prevention, give misoprostol PO: 600 µg after the delivery of the baby (or Oxytocin 1IU IM/IV). If an IV infusion is in place, Oxytocin IV 10 IU may be given as a slow push. *Note: the*

potential side effects of misoprostol include fever, chills, nausea vomiting and diarrhea, similar to Ebola symptoms. However, misoprostol related side effects are often self-limiting. Provide care accordingly.

b. For treatment: 2 IV lines with 1 L of Ringers, one with Ringers oxytocin 40 IU and let run over 4 hours. In addition to fluids for prevention and management of hypovolemic shock, infuse 40 units of oxytocin in 1 L - run it in initially at 60 drops per min while uterus contracts, then lower infusion rate to 40 drops per min. Add Misoprostol PO 600 µg (= 3 tabs, preferably oral if the patient can swallow, otherwise per rectum) when not given as prevention.

c. If feasible, perform external uterine massage with protective covering (such as blue pads/adult diaper underpads) on the patient's abdomen, while standing on the side of the patient. To avoid exposure to blood and other body fluids do not face the patient directly**No suturing of cervical nor vaginal tears**; the risk for a needle stick injury is too high.

d. **No bimanual compression of the uterus** in order to avoid blood contact (or any other intrauterine maneuver).

2. **RETAINED PLACENTA (pieces):**

Continue prophylactic antibiotics until the placenta is delivered. NO MANUAL REMOVAL OF PLACENTA OR UTERINE REVISION. Trial of oxytocin 20 IU in 1 L of Ringers over 12 hours. Misoprostol PO 800 µg (4 tablets) can also be given as an alternative (orally is here preferred, rectal administration is possible if the patient cannot swallow).

3. **RETAINED PRODUCTS OF CONCEPTION (after miscarriage):**

GIVE Misoprostol 800µg oral x 1; and can be repeated after 48 hours if no expulsion of retained products **No manual vacuum aspiration** (MVA), dilatation & curettage (D &C), or digital curettage.

4. **COMPLICATIONS REQUIRING SURGERY CANNOT BE TREATED** (cephalopelvic disproportion, malpresentation, ruptured uterus, ectopic pregnancy, molar pregnancy, abruption of placenta with no imminent delivery). NO BLOOD TRANSFUSIONS in the ETC.

Waste Management

- Decontaminate placenta and blood products with 0.5 % chlorine before disposal;
- Put the placenta in a thick plastic bag and add 0.5% chlorine solution. This should be placed in another bag (double bagged) spraying the bag with 0.5% chlorine solution;
- Dispose of the placenta in the placenta in the black double plastic, sprayed and brought to the burning pit.
- DON'T give placenta to family members, counsel them about the risk of infection
- Almost all babies born in the ETC will be stillborn, the corpse and placenta can be treated in the following way: placenta and corpse together on (absorbable) cloth; chlorine spraying, then wrapped in more cloth, spraying, then in a (child) body bag.

Newborn/Infant Feeding

1. Feeding of an alive born child in ETC:

Since Ebola is transmitted across the placenta, the possibility of survival of the baby is almost zero. In the very rare event the baby is being born alive: if the mother is able to breastfeed, the best recommendation is to initiate breastfeeding. If the mother is not able to breastfeed, the baby is considered contagious, admit the baby with the mother and offer formula milk for this baby until the survival of the mother is clearer. Explain to the family that the baby is a potential source of Ebola transmission.

2. Feeding of Ebola negative children from breastfeeding mothers admitted to the ETC

When a woman is admitted who is still breastfeeding, the child should no longer receive breast milk at this

stage and should be separated from the mother. Further, the child will have been exposed to body fluids of an Ebola-infected mother during the first stages of the illness; there for, most children should be tested as well. Also, the child is a risk for the other family members/friends to contract Ebola if they would breastfeed this child. Therefore wet- nursing is not recommended.

a. For children of more than 6 months of age: stop breastfeeding, the child should be weaned. Give formula milk and/or plumpy-nut for the transition period to other food (several weeks).

b. For children of less than 6 months of age: stop breastfeeding, formula feeding should be provided till the age of 6 months, then, other foods can be introduced.

When stopping abruptly with breastfeeding, the mother will need to express her milk to alleviate pain and prevent inflammation. Women admitted to the ETC who have established breastfeeding should receive a personal breast pump or shown how to express milk manually, in order to prevent breast engorgement unless her clinical condition reduces already spontaneously the milk production. Practically this means: one pump per patient while they are admitted in the ETC, burned afterwards and another one at moment of discharge if needed. In the ETC, expressed milk will be discarded in a bucket with chlorinated water. At home, women should discard the milk into the latrine. It is important to explain this to women when discharged from the ETC.

If the mother survives Ebola, the virus is still present in the breast milk, even when the viraemia in the blood is negative. At the moment it is not known for how long this is. It is therefore not recommended to resume breastfeeding (unless testing of the milk has confirmed the absence of the virus in the milk).

5. Feeding of Ebola positive children of breastfeeding mothers admitted to the ETC

Continue breastfeeding

Instruction on How to Use Ready to Use Infant Formula (RUIF) for Infants 0-12 Months

Key Message

Exclusive Breastfeeding is the best way to feed an Infant under 6 Months of Age; however, the safest replacement feeding option in the current context is RUIF.

What is Ready-to Use Infant Formula (RUIF)?

- RUIF is specially constituted, nutritionally complete breast milk substitute.
- It is a liquid pure milk that has been treated with Ultra high temperature to destroy the microorganisms and preserve the milk to prevent it from being spoilt.
- Does not require warming, measuring or mixing with water just open and serve; therefore, reduces risk of cross contamination.

How to use Ready to Use Infant Formula (RUIF)

- Clean the preparation surface, for example the lid of storage container
- Wash hands before opening the box
- Shake well before opening
- Use a clean knife or scissors to open the RUIF packaging
- Pour in required quantity of RUIF into a clean cup
- RUIF can be fed in room temperature, if required to heat place the cup in warm water
- Infants 0-5 months should be fed 6-8 times (or more) per day (please see below quantities per feed for each age)
- Infants 6-12 months should be fed 3-4 times (or more) per day, each fed up to 200ml (2 disposable cups)

Age of Infant in Months	Amount of formula per day**	Number of feeds per day	Size of feed in ml
0-1	450ml	8	60ml
1-2	600ml	7	90ml
2-3	750ml	6	120ml
3-4	750ml	6	120ml
4-5	900ml	6	150ml
6-8	900ml	5	180ml
9-12	800ml	4	200ml

**The amount of milk per day varies depending on amount of food the baby is able to eat per feed

Food Hygiene

- Remember to always wash had with soap and water before handling the milk, before and after feeding the infant
- Throw away any remaining ready-to-use infant formula within two hours.

Who should consume Ready-to Use Infant Formula (RUIF)?

- Infants 0-12 months whose mother are infected with EVD in treatment or holding or interim care centers
- Infants 0-12 months in Ebola treatment centers

How to feed RUIF to infants 0-12 months

- Clean the cup with hot, preferably boiling, water.
- Hold the child in semi-upright position
- Do not pour the milk directly into the baby's mouth
- The infant will take up the milk with their tongue, suck or sip.
- If the baby can sit and hold the cup by themselves allow them to drink by themselves
- Encourage the infant to feed by cup
- Dispose the cup after every feed

NOTE: for those **children aged 6-12 months** who have **regained appetite** adequate amounts of appropriate and hygienically prepared **complementary food** should be provided in addition to milk

NOTE: for holding and treatment centers disposable cups must be used for feeding infants and disposed after every use

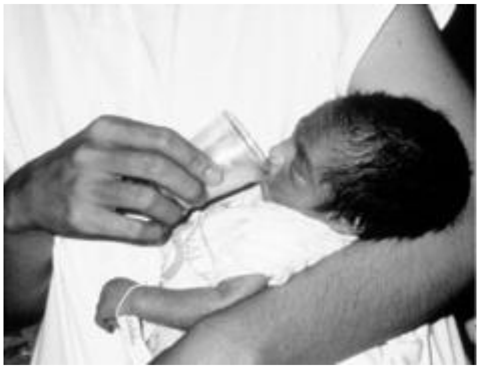

Discharge of Pregnant/Post-Delivery/Post Miscarriage Women

DISCHARGE OF A SURVIVOR WHO IS STILL PREGNANT:

After the second confirmed negative test, a woman who is still pregnant should be referred to specialized care for further management.

DISCHARGE POST DELIVERY OR POST MISCARRIAGE:

Discharge criteria apply as for other patients admitted to the ETC (negative viraemia and good clinical status).

The only exception is for pregnant women: once the viraemia is negative, induction can be done and delivery should still take place in the ETC because of the amniotic fluid and placenta that will still be Ebola positive.

1. After delivery and an observation period of 24 hours, the woman can be discharged from the ETC. If the baby is born alive, the baby should be kept in the ETC with the mother and should be breastfed.
2. Extra hygienic pads can be given. Explain to the woman that she must discard the pad into a plastic bag and to burn it.
3. Family planning: Every woman whether or not she has been pregnant who survived Ebola should be offered family planning, at least 3-6 months (implants, pills, injections). Refer to local facility for FP.
4. Give Iron/Folate for 1 month to women who had a miscarriage or delivery.
5. Advise on condom use and provide condoms for 3 months
6. Advise that she MUST NOT wet nurse another infant.

Psychosocial Support

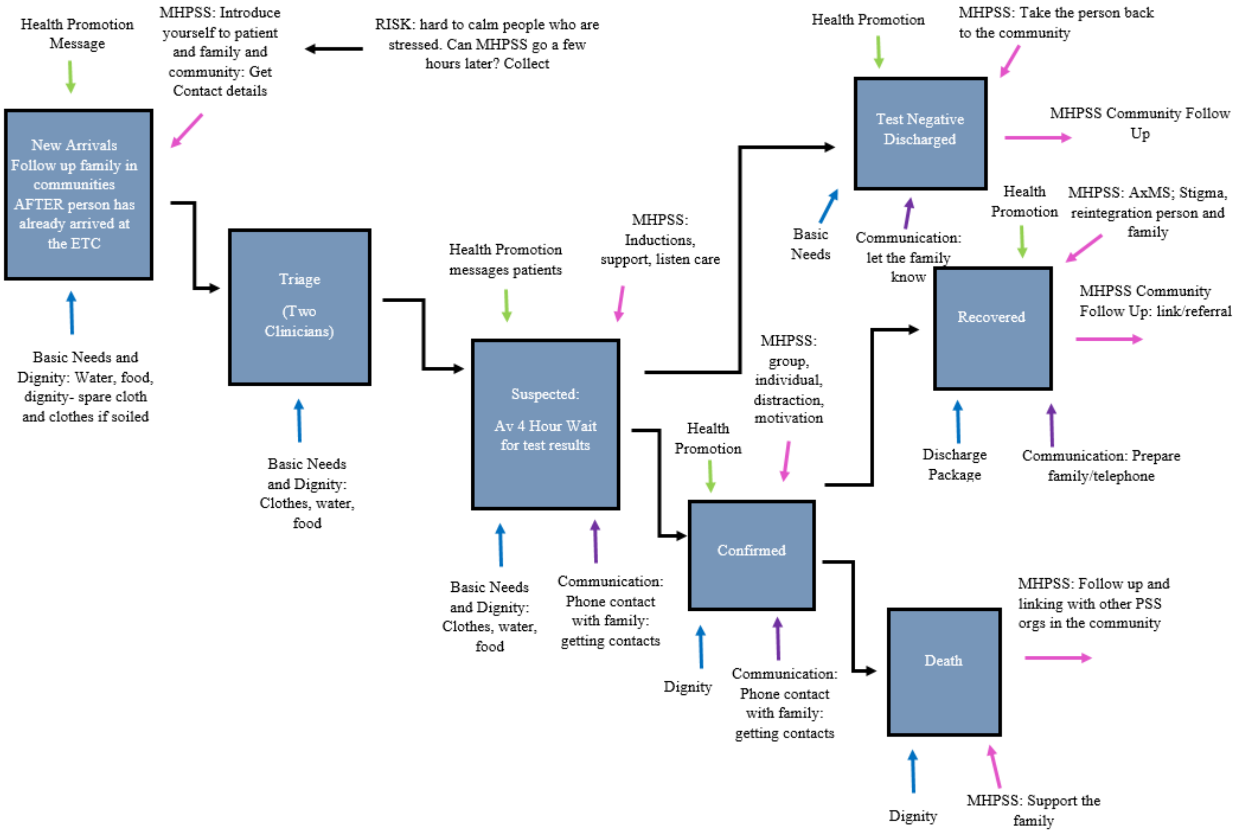

Admissions Protocol – Initial psychosocial contact

If patient arrives at the treatment center with family members then the patient goes to Triage and the family are given the contact details for the PSS ETC team and encouraged to go home and wait for the District Command Surveillance team to visit as they are contacts and need to be monitored for 21 days.

(Psychosocial team to stay in contact with family during this time) Psychosocial Team:

Allocation.

1 PSS officer allocated to each new patient.

Allocated PSS officer to liaise with Triage nurses and the nursing team to find out basic details, physical health, risks, contact details for the person and their family.

Allocated PSS Officer to introduce themselves to the family in the Family Area while patient is triaged and admitted.

This is a potential risk area: Abide by the infection control protocols; do not touch the family members only sit in the Blue Chairs and ensure Family only sit in the grey chairs; Keep a safe distance of 3 foot or one meter. Aim of this interaction is to relieve distress of the family, orientate them to the site, deliver health promotion messages, deliver do's and don'ts of the ETC. IF the family wants to talk then allow them to talk about what happened but do not force them (Use principles of PFA)

Allocated PSS officer to make contact **within one hour** of the patient entering the suspected of confirmed ward

IF PATIENT IS WELL ENOUGH TO COME OUTSIDE:

Allocated PSS officer should introduce themselves to their allocated patients

Aim of the introduction is to relieve distress, offer some calm support, identify yourself, try and understand how the person is feeling and what is happening for them IF they are willing to talk then take down some basic information (see assessment sheet)
Give them space to talk if they want BUT do not force them to talk. If they do not want to talk then let them know that you are available for them and will come back later.

IF THE PATIENT IS TOO UNWELL TO COME OUTSIDE:

PSS officer must liaise with the medical team to understand more about the presentation of that patient on the ward.

If the patient is presenting in a very low mood but are physically able to leave their bed area then the medical team should encourage them to come outside and talk to PSS.

ADMISSIONS PROTOCOL FOR UNACCOMPANIED CHILDREN

If an unaccompanied child is admitted to the ETC the allocated PSS team must inform the Representative from the Ministry of Social Welfare and UNICEF (OR other Family Tracing and Reunification Partners) at Incident Command Centre.

Follow-up: 2nd Psychosocial contact

The second Psychosocial Contact must be the same day.

EITHER in the form of:

Individual sessions - Approach the person for a second time if they did not want to talk to you earlier (If they

still do not want to talk to you that is fine DO NOT FORCE THEM)

Individual sessions if the patient requests it OR

Group Sessions: As a team PSS Officers will be running at least 2 group sessions per day, every day for the patients.

IF THE FAMILY OF THE PATIENT ARE PRESENT:

PSS should help the family to walk round to the family visiting area so that they can see their loved ones.

IF THE FAMILY ARE NOT PRESENT

PSS team to contact patients family by mobile phone OR community outreach visit to engage the family and reduce distress in the communities.

Continuing Care: Follow up during the stay in the ETC

Allocated PSS Officers must have one meaningful conversation per day, every day with their patients.
Allocated PSS Officers must prioritize communication with families and communities Allocated PSS Officers must run at least 2 different groups every day.
At night PSS to run films for the patients

Psychosocial Discharge protocol

Receive 48 hours notice from Clinical Team after 1st negative blood test of the patient.

At **48 hour point** notify MOSWG, SW and MHPSS partners of discharge patient and put into place actions to accommodate and welcome back the survivors.

In the case of children we need to inform MOSWG (they were informed at the point of admission so should have already been tracing family) that the child is ready for discharge and ensure Family Retracing has been done and/or accommodation for the child has been found.

Family contacted to prepare the family and community for reintegration

At 48-24 hours PSS team offer psychosocial discharge interview to identify patients needs and coping strategies and create a realistic plan as to how the person can cope and what to expect in the community.

Organize transport for the patient for the next day depending on distance required to travel.

Day of discharge: Explanation of what is in the discharge package from IMC and the Discharge package from WFP (what they can expect and when).

After patient has washed and changed clothes discharge event to celebrate the person’s future.

Burial

When patients pass away, the PSS team facilitates family contact, family support to view the body in the morgue, and work closely with burial teams to ensure that the families are satisfied with the way their loved ones were treated in death. The PSS team also facilitates continued communication with the family as needed and visitation of burial sites.

ANNEX I

Laboratory/IV Procedure

Laboratory Testing

1. All patients will have Ebola blood test done on arrival in a EDTA (purple top) tube
2. Repeat Ebola blood test to be done after clinical improvement AND 3 days without fever or any other Ebola symptoms. If positive, this is repeated every 48 hours until negative
3. If the lab reports a result as indeterminate (+/-) additional comments:
    a. Keep the patient in whichever ward they are presently located
    b. If unwell suspect/probable, retake blood at the next opportunity
    c. If well (all wards): retake blood 24 hours later
    d. Where possible take pediatric blood via cannula/butterfly as an EDTA sample. If the initial pediatric sample was a blood swab, use EDTA sample for any repeat testing.
4. Mouth swabs are **ONLY** to be used after death

Invasive Procedures

For drawing blood and starting IV Fluids

- Clinician to identify patient. Prepare the equipment for taking specimen. Label specimen bags and vials with indelible ink. All of these items can be obtained from pharmacy and must be prepared before entry into the high-risk zone
- All patients (suspect/probable) must have their blood specimen obtained following triage. This enables a speedier confirmation of their Ebola status, which in turn allows us to support patients better.
- All clinicians must ensure that they are competent and able to perform this clinical task. All equipment must be to hand before the procedure starts and must include a sharps bin, needles, mini chlorine sprayer etc. All sharps must be disposed of safely into a sharps bin following completion of the procedure.
- Clearly label the tubes and the packaging (clear Ziploc bags)
- Clearly label IV solution with patient's name as well as contents and flow rate
- Prepare the drawing device with the needle capped, the disinfecting swab, a dry gauze tape for occluding the puncture site and the glove for tourniquet
- Make sure the second packaging is ready at the transition zone to receive your Ziploc bag with samples already sprayed
- Draw blood samples and do all procedures at the beginning of your time in the high risk zone. The procedure might take more time than you expect!
- Confirm the identity of each patient before each procedure by asking his/her name and checking their wrist band
- If you draw blood or start IV lines, your colleague should be in charge of spraying
- Spray the place where you will sit (usually a stool) and install yourself comfortably, if possible keeping your distance from the head of the patient.
- Spray the inside of the Ziploc bag. Empty any remaining chlorine solution and keep it

ready to receive the sample

- Make sure the sharp box is handy,
- Always explain to the patient and to your colleague what you are about to do
- Ask the patient to keep pushing on the puncture site (sick patients might bleed a lot) while you spray the tube before dropping it in the sprayed Ziploc bag
- Tape the gauze on the arm and ask the patient to keep pushing on it for 10 minutes (if able)
- Spray your gloved hands every time you have touched the patient
- Spray the outside of the Ziploc bag containing the sample
- Once your sample is taken, or IV is started, immediately dispose of the needle in the sharp box
- Go to the transition zone (doffing area) and drop your folded Ziploc bag into the second Ziploc bag or container. It must then be brought to the doffing area where it must be put in a bucket of chlorinated water of 0.5% concentration for half an hour only.
- The clinician is then responsible for making sure that the specimen is transferred to pharmacy safely in a receptacle to be stored in the blood specimen fridge until collection can be arranged.
- Pharmacy staff should make contact with the transport manager and ensure the specimen is contained in the appropriate container before carrying it to the vehicle.
- Triple packaged samples will be transported in a bucket with a lid clearly identified to the laboratory or kept in a locked fridge (depending on the place)
- For IV starts:
    - Connect IV tubing once sharp is placed in sharps container
    - Tape IV catheter securely in place
    - Set flow rate of IV fluid/medication

ANNEX II

IMC Sierra Leone Ebola Treatment Center
MANAGEMENT OF MALERIA

All patients admitted in suspected, probably or confirmed wards must receive antimalarial treatment with either Coartem (if able to take oral medications) or treated for severe malaria if unable to take oral medications or meet **one or more** of the following criteria:

Check for signs of severe malaria:

- Prostration (unable to walk/sit without support or drink/breastfeed)
- $\geq$ 2 convulsions in 24 hours
- Altered consciousness
- Shock
- Increased work of breathing where pneumonia is unlikely

One or more signs present →

Assume Severe Malaria: Treat with Artemether IM (preferred) or Artesunate IV/IM

1. Use dosage chart
2. Artemether:
   Peds: Loading dose 3.2 mg/kg, then maintenance dose 1.6 mg/kg (daily).
   Adults: 160 mg loading dose, then 80 mg maintenance dose (once daily).
   Use a minimum of loading dose and one maintenance dose before switching to oral treatment (Coartem)
   Artesunate: 2.4 mg/kg at time 0 hr, 12 hr, 24 hr, then once daily (see preparation instructions below)
3. If the patient is unable to tolerate oral medication continue injection drugs for children: 6 days; for adults: 4 days (Artemether), 6 days (Artesunate)
4. Treat co-infection: give Ceftriaxone according to dosage chart x total 7 days

Artemether: If improved after 1 loading dose and 1 maintenance does and able to take oral treatment: Change to Coartem x 3 days

Artesunate: If improved, use a minimum of 3 doses before switching to oral treatment with Coartem x 3 days

1. Use dosage chart
2. Continue to treat any co-infectious: switch to Cefixime once daily to complete 7 days

↓ Signs Absent AND able to take oral medications

Uncomplicated Malaria: Treat with Coartem

1. Use dosage chart
2. Treat any co-infectious: give Cefixime po once daily x 5 days

	Artemether: Loading 3.2 mg/kg Maintenance dose 1.6 mg/kg (once daily)		Artesunate: Dose 2.4mg/kg at time 0 hr, 12 hr, 24 hr, then once daily			
	IM in thigh		IV		IM	
**Weight (kg)**	**Loading dose (mg)**	**Maintenance dose (mg) once daily**	**Dose (mg)**	**Dose (mL) of 60mg in 6 mL**	**Dose (mg)**	**Dose (mL) of 60mg in 6 mL**
3.0 – 3.9	10	5	7.5	0.75	10	1
4.0 – 4.9	13	6	10	1	10	1
5.0 – 5.9	16	8	12	1.2	20	1
6.0 – 6.9	19	10	15	1.5	20	1
7.0 – 7.9	22	11	17	1.7	20	1
8.0 – 8.9	26	13	19	1.9	20	1
9.0 – 9.9	29	14	22	2.2	30	2
10.0 – 10.9	32	16	24	2.4	30	2
11.0 – 11.9	35	18	26	2.6	30	2
12.0 – 12.9	38	19	29	2.9	30	2
13.0 – 13.9	42	21	31	3.1	40	2
14.0 – 14.9	45	22	34	3.4	40	2
15.0 – 15.9	48	24	36	3.6	40	2
16.0 – 16.9	51	26	38	3.8	40	2
17.0 – 17.9	54	27	41	4.1	50	3
18.0 – 18.9	58	29	43	4.3	50	3
19.0 – 19.9	61	30	46	4.6	50	3
20.0 – 20.9	64	32	48	4.8	50	3

Artemether IM: For weights above 21 kg, calculate dosage according to mg/kg dosages in chart above (with maximum of dose of 160mg (loading dose), 80 mg (maintenance dose)

Preparation/use of Artesunate IV/IM

Give every 12 hours for the first three doses (time 0, 12 hr, and 24 hr). Use a minimum of 3 doses before switching to oral Coartem. If unable to tolerate oral medications, continue at 2.4mg/kg once daily for a maximum of 5 more days.

Artesunate typically comes as a powder together with a 1 mL vial of 5% bicarbonate that then needs to be further diluted with either Ringers Lactate or 5% Dextrose

- DO NOT use water for injection to prepare artesunate for injection
- DO NOT give artesunate if the solution in the syringe is cloudy
- DO NOT give artesunate as a slow IV drip (infusion), GIVE by bolus over 1 minute
- YOU MUST use artesunate within 1 hour after it is prepared for injection

Preparing IV Artesunate	IV	IM
Artesunate powder (mg)	60mg	60mg
Ringers Lactate or 5% Dextrose (mL)	5mL	2mL
Artesunate concentration mg/mL	10mg/mL	20mg/mL

ANNEX III

PEDIATRIC FLUID MANAGEMENT GUIDELINES

Children usually tolerate oral fluid very well but if IV fluids are required please follow below guidelines. An assessment of nutritional status must be done prior to giving IV fluids to determine fluid management pathway.

- If severely malnourished (Z-score < -3 SD for all children) refer to fluid management protocol for malnourished children

1. MAINTENANCE FLUIDS

When to give maintenance fluids:
If not in shock or severe dehydration, manage patient via oral or NG tube fluids.

If unable to meet fluid replacement targets (not tolerating oral fluids, or are unable to take adequate volume by mouth, or have significant ongoing losses) consider starting on maintenance IV fluids.
For children under 5, consider staring IV fluids once Ebola positive status is confirmed due to the possibility of rapid deterioration.

Which fluids to give as maintenance:
<1 month – ½ Hartmanns (or ½ Ringer's Lactate) + D10 (or ½ Normal Saline + D10)

≥1 month – ½ Hartmanns (or ½ Ringer's Lactate) + D5 (or ½ Normal saline + D5)

Refer to fluid table below for pre-calculated volumes and rates of maintenance fluids.

2. BOLUS FLUIDS

If they are severely dehydrated or in shock
(Please see classification guideline if you are unsure how to assess this)

Which fluid to give:
This is based on their nutritional status (Based upon Z-score done on triage. Please see guideline):

- **Well-Nourished: Ringer's Lactate (RL) or Hartmann's 20ml/kg x1 over 20mins**
- **Severely Malnourished: D5 in Ringer's Lactate or Hartmann's 15ml/kg over 1 hour**

IV Fluid Choice for Management of Shock/Severe Dehydration

Condition	Age	Give Initial (bolus)	Then start maintenance
Shock/severe dehydration **without malnutrition**	< 1 month	RL or Hartmann's	10% Dextrose + 1/2 Hartmann's (or RL)
	≥ 1 month		5% Dextrose + 1/2 Hartmann's (or RL)
Shock/severe dehydration in children **with severe malnutrition**	< 1 month	5% Dextrose + Hartmann's or RL	10% Dextrose + 1/2 Hartmann's (or RL)
	≥ 1 month		5% Dextrose + 1/2 Hartmann's (or RL)

If no IV access able to be obtained or if lose IV access:

- Consider placing IO if appropriately skilled clinician available and use IO for initial fluid resuscitation. IO must be removed after a maximum of 24 hours and another route used.
- Place NG tube (if no bleeding or vomiting) and do fluid resuscitation via NG tube. ALWAYS aspirate small volume of gastric contents before placing any fluid down the NG Tube. If no fluid able to be aspirated, push small amount of air through and feel over stomach for air movement to confirm NG tube placement.

Following initial fluid resuscitation aim to establish IV access if possible.

Please see management of dehydration table for further action following initial bolus.

PREPARATION OF DIFFERENT TYPES OF FLUIDS

½ Ringers Lactate and D5: (This is the same as ½ Ringers Lactate and ½ D10)
Firstly make 1 liter of D10:

- Take 1 liter of D5, remove 111mls and replace it with 111mls of D50 This gives you 1 liter of D10. THEN:
- Take a 500ml bag of Ringers Lactate and remove 250mls
- Take 250mls of the D10 you have made and add it to the remaining 250mls of Ringers Lactate. **This makes 500ml of ½ Ringers Lactate and D5**

½ Ringers Lactate and D10
Firstly make 1 liter of D20:

- Take 1 liter of D5, remove 333mls and replace it with 333mls of D50 This gives you 1 liter of D20. THEN:
- Take a 500ml bag of Ringers Lactate and remove 250mls
- Take 250mls of the D20 you have made and add it to the remaining 250mls of Ringers Lactate **This makes 500ml of ½ Ringers**

D5 in Ringers Lactate

- Remove 50 ml of fluid from 500ml Ringers Lactate
- Add in 50 ml of 50% Dextrose **This makes 500ml of D5 in Ringers Lactate**

Preparing IV Medications
All of the following should be diluted to 1mg/1ml with normal saline and the requested dose should be provided:

- **Morphine** (10mg/1ml preparation)
- **Ondansetron** (2mg/1ml preparation)
- **Diazepam** (5mg/1ml preparation)

For Example:
A prescription for 5mg of IV morphine:

- Dilute 10mg of IV morphine with 9mls of normal saline to make 10mls of 1mg/1ml IV morphine
- Then give 5mls to provide the requested 5mg of IV morphine **Please provide an IV flush for all sets of IV medication.**

Oral Fluid Management

Oral Rehydration Solution:
Targets: 40ml/kg/day without dehydration, 80ml/kg/day with moderate dehydration

Weight (KG)	**Child without dehydration** 40 mls/kg/day		***Child with moderate dehydration** 80mls/kg/day	
	**Total Volume/day**	**Target volume every 6 hours**	**Target volume / day**	**Target volume every 6 hours**
< 5	200 mls	50 mls every 6 hrs	400 mls	100 mls every 6 hrs
6-9	320 mls	80 mls every 6 hrs	640 mls	160 mls every 6 hrs
10-14	500 mls	125 mls every 6 hrs	1000 mls	250 mls every 6 hrs
15-19	750 mls	180 mls every 6 hrs	1,500 mls	375 mls every 6 hrs
20-24	950 mls	230 mls every 6 hrs	1,900 mls	475 mls every 6 hrs
25-29	1000 mls	250 mls every 6 hrs	2000 mls	500 mls every 6 hrs
30-34	1300 mls	325 mls every 6 hrs	2,600 mls	650 mls every 6 hrs
35-39	1500 mls	375 mls every 6 hrs	3,000 mls	750 mls every 6 hrs
40-44	1500 mls	375 mls every 6 hrs	3,000 mls	750 mls every 6 hrs
45-49	1500 mls	375 mls every 6 hrs	3,000 mls	750 mls every 6 hrs
50	1500 mls	375 mls every 6 hrs	3,000 mls	750 mls every 6 hrs

*If unable to reach targets and/or large volume of loss (diarrhea), consider placing NG tube (if no bleeding or vomiting) or starting IV fluids at maintenance

Management of shock/severe dehydration in Children

Signs of shock in children:

1. Cold hands **PLUS**
2. Weak or absent pulse and **EITHER**
3. Capillary refill > 3 seconds **OR**
4. AVPU less than Alert

Children in shock who require bolus fluid resuscitation are often lethargic and have cold skin, prolonged capillary refill, fast weak pulse and hypotension.

- Check whether the child's hand is cold. If so, determine whether the child is in shock.
- Check weather the capillary refill time is longer than 3 seconds.
- If capillary refill is longer than 3 seconds, check the pulse. Is it weak and fast? If the radial pulse is strong and not obviously fast, the child is not in shock. If you cannot feel the radial pulse of an infant < 1 year old, feel the brachial pulse or if the infant is lying down, the femoral pulse. If you cannot feel the radial pulse of a child, feel the carotid

Normal pulse rate and respiratory rate in children

Age (years)	Pulse Rate (range)	Respiratory Rate
0-1	100-160	0-3 months: 35-55<br>3-6 months: 30-45<br>6-12 months: 25-40
1-3	90-150	20-30
3-6	80-140	20-25

Note: normal pulse rates are 10% slower in sleeping children.

IV Fluid Management: Severe Dehydration/Shock
Children without Severe Malnutrition

Weight (kg)	Initial bolus: 20 ml/kg Hartmann's or Ringer's Lactate
	Volume (mls)
2	40
3	60
4	80
5	100
6	120
7	140
8	160
9	180
10	200
11	220
12	240
13	260
14	280
15	300
16	320
17	340
18	360
19	380
20	400

<u>Hydration Targets:</u> Improved mental status, normal pulse, and warm extremities. Can repeat 20 mg/kg x 1 if no improvement. Then continue IV fluids at maintenance until able to tolerate appropriate oral fluids for age.

IV Fluid Management in Children with Severe Malnutrition

Urgent Fluid management – Child WITH severe malnutrition<br>5% dextrose in Hartmann's or Ringer's Lactate			
	**Shock**<br>**Initial bolus: 15 ml/kg IV**		**Oral / NG tube Resomal**<br>**10 ml/kg/hr**
**Weight (kg)**	**Volume (ml) over I hour**	**Drops/min (If 20 drops/ml giving set)**	**Target volume (ml) every 6 hours for 12 hours**
3	45	15	180 ml
4	60	20	240 ml
5	75	25	300 ml
6	90	30	360 ml
7	105	35	420 ml
8	120	40	480 ml
9	135	45	540 ml
10	150	50	600 ml
11	165	55	660 ml
12	180	60	720 ml
13	200	65	790 ml
14	220	70	840 ml
15	240	80	900 ml

Hydration Targets: Improved mental status, normal pulse, and warm extremities. If improvement, repeat bolus x1, then advance to Resomal oral/NG fluid and after 12 hours continue ORS & add F75 per standard protocol. Offer food as tolerated. If no improvement after initial bolus, give maintenance fluids at 4ml/kg/hour.

Maintenance IV Fluid Management in Children

For children ≥ 1 month of age use Dextrose 5% + ½ Ringers Lactate<br>For neonates (< 1 month of age) use Dextrose 10% + ½ Ringers Lactate				
**Weight (kg)**	**Total volume (mls)/day Maintenance**	**Drops/min**	**Total volume (mls)/day 1.5 x Maintenance**	**Drops/min**
2	200	3	300	4
3	300	4	450	6
4	400	6	600	8
5	500	7	750	10
6	600	8	900	13
7	700	10	1000	14
8	800	11	1250	17
9	900	13	1250	17
10	950	14	1500	20
11	1000	14	1500	20
12	1000	14	1500	20
13	1000	14	1500	20
14	1250	17	2000	28
15	1250	17	2000	28
16	1250	17	2000	28
17	1250	17	2000	28
18	1250	17	2000	28
19	1500	21	2250	30
20	1500	21	2250	30
21	1500	21	2250	30
22	1500	21	2250	30
23	1500	21	2250	30
24	1500	21	2250	30
25	1500	21	2250	30
26	1500	21	2250	30
27	1500	21	2250	30
28	1500	21	2250	30
29	1650	23	2500	35
30	1650	23	2500	35

MANAGEMENT OF FLUID OVERLOAD:

Monitor for fluid overload

Fluid overload is an important complication of treatment for shock/severe dehydration due to excess or too rapid IV fluid administration or use of large volume of fluid in children with severe capillary leakage (as seen in EVD).

Look for:

- Fast breathing
- Chest indrawing
- Periorbital or soft tissue edema
- Ascites

Management:

Furosemide: 1-2 mg/kg IV twice daily **(maximum pediatric dose 2 mg/kg/dose, maximum adult dose 8mg/dose)**

Weight	1 mg/kg IV of 10mg/ml formulation	2 mg/kg IV of 10 mg/ml formulation
3 to < 6 kg	0.4 ml	0.8 ml
6 to < 10 kg	0.8 ml	1.6 ml
10 to < 15 kg	1.2 ml	2.4 ml
15 to < 20 kg	1.7 ml	3.4 ml
20 to < 29 kg	2.5 ml	5 ml
